# Tissue‐resident mucosal‐associated invariant T (MAIT) cells in the human kidney represent a functionally distinct subset

**DOI:** 10.1002/eji.202048644

**Published:** 2020-08-06

**Authors:** Matty L. Terpstra, Ester B.M. Remmerswaal, Nelly D. van der Bom‐Baylon, Marjan J. Sinnige, Jesper Kers, Michiel C. van Aalderen, Suzanne E. Geerlings, Frederike J. Bemelman

**Affiliations:** ^1^ Division of Internal Medicine Department of Nephrology Renal Transplant Unit Amsterdam Infection & Immunity Institute (AI&II) Amsterdam UMC University of Amsterdam Amsterdam The Netherlands; ^2^ Department of Experimental Immunology Amsterdam Infection & Immunity Institute (AI&II) Amsterdam UMC University of Amsterdam Amsterdam The Netherlands; ^3^ Department of Pathology Amsterdam Infection & Immunity Institute (AI&II), Amsterdam UMC University of Amsterdam Amsterdam The Netherlands; ^4^ Center for Analytical Sciences Amsterdam (CASA)—Biomolecular Systems Analytics Van‘t Hoff Institute for Molecular Sciences (HIMS) University of Amsterdam Amsterdam The Netherlands; ^5^ Department of Pathology Leiden University Medical Center Leiden The Netherlands; ^6^ Department of Internal Medicine Infectious Diseases Amsterdam Infection & Immunity Institute (AI&II), Amsterdam UMC University of Amsterdam Amsterdam The Netherlands

**Keywords:** MAIT cells, renal transplantation, tissue‐residency

## Abstract

Mucosal‐associated invariant T (MAIT) cells are innate‐like T‐cells that recognize bacterial riboflavin metabolites. They are present in human blood but are abundant at barrier sites, including the liver, lungs, and kidneys, where they possess a CD69^+^/CD103^+/−^ tissue‐resident phenotype. In renal tissue, MAIT cells likely defend against the ascending uropathogens responsible for urinary tract infections (UTIs), which are common, especially among renal transplant recipients (RTRs). Nevertheless, the functional role for MAIT cells in renal tissue and the influence of renal transplantation on MAIT cells remains unclear. Using multiparameter flow cytometry and the MR1‐tetramer, we characterized MAIT cell phenotype and function in healthy renal tissue (n = 6), renal transplants explanted after allograft failure (n = 14) and in blood from healthy controls (n = 20) and RTRs before and 1‐year after transplantation (n = 21). MAIT cells in renal tissue constitute a distinct CD69^+^CD103^+/−^ population that displays typical phenotypic features of tissue‐resident T‐cells and is skewed toward IL‐2, GM‐CSF, and IL‐17A production upon stimulation. The circulating MAIT cell population was not decreased in number in RTRs pre‐ or post‐transplantation. Tissue‐resident MAIT cells in the kidney represent a functionally distinct population. This shows how MAIT cells in the kidney may be involved in the protection against microorganisms.

## Graphical abstract

MAIT cells, innate‐like T‐cells involved in the antibacterial response, have recently been identified for the first time in renal tissue. Our results show that MAIT cells in the kidney express a distinct tissue‐resident phenotype and possess specialized local memory functions such as the production of immune response enhancing cytokines.

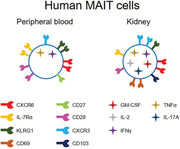

## Introduction

Mucosal‐associated invariant T (MAIT) cells are innate‐like T‐cells involved in the antibacterial and antifungal response by recognizing riboflavin metabolites. They comprise 0.1‐10% of the T‐cell population in the peripheral blood and are abundant at barrier sites such as the liver, lungs, intestine, stomach, and the female genital mucosa [1, 2, 3, 4, 5, 6]. MAIT cells are characterized by the expression of the semi‐invariant Vα7.2‐Jα12/20/33 chain of the TCR, which restricts them to the nonpolymorphic, MHC class I‐related protein MR1 [7, 8, 9, 10]. MR1 presents unstable pyrimidine intermediates derived from the riboflavin biosynthesis pathway used by most bacteria and some fungi, but not humans [11, 12, 13].

Until recently, MAIT cells were defined by expression of Vα7.2^+^ CD161^++^, which may be unreliable due to CD161 downregulating after antigen stimulation [14]. The development of the MR1 tetramer enables the highly specific detection of MAIT cells based on their TCR configuration [6, 15, 16]. Because MAIT cells respond to a wide range of bacteria, among which *Escherichia coli*, their importance to microbial immunity is increasingly recognized. MAIT cells can accumulate at the site of infection and have been proven to be protective in various experimental infection models [9, 17, 18]. In an experimental mouse model of urinary tract infection (UTI), MAIT cells were shown to migrate to the bladder, where they decreased the bacterial load [19]. Recently, MAIT cells were also shown to reside in the human kidney. These cells were found to express CD69 (a C‐type lectin) and CD103 (the integrin αE chain), marking them as tissue‐resident T cells [5].

UTIs are among the most common infection worldwide [20, 21], and immunocompromised patients, such as renal transplant recipients (RTRs) and patients with end‐stage renal disease (ESRD), are particularly vulnerable to UTIs [22, 23]. One of the factors underlying this enhanced vulnerability for UTIs in RTRs might be a decreased number of MAIT cells in circulation, as was recently shown both in patients with ESRD and RTRs years after transplantation [24, 25, 26, 27]. However, little is known about the phenotype and function of (tissue‐resident) MAIT cells in healthy human kidneys or how they are affected by kidney transplantation.

Therefore, we performed an extensive phenotypic and functional characterization of MAIT cells from healthy renal tissue, explanted renal allografts, and from the circulation of healthy individuals and RTRs before and 1‐year after transplantation. These analyses revealed that MAIT cells in renal tissue represent a distinct CD69^+^CD103^+/‐^ tissue‐resident population that display the typical features of tissue‐resident T‐cells such as a decreased percentage of cells expressing KLRG1 and an increased percentage of cells expressing CXCR6 compared to nontissue resident (CD69^−^CD103^−^) cells. Upon stimulation, CD103^+^ kidney‐derived MAIT cells secrete IL‐2, GM‐CSF, and IL‐17A, which indicates that they exert local memory functions distinct from those of their circulating counterparts. These findings fit with a role for tissue‐resident MAIT cells as the first line of defense against invading pathogens.

## Results

### MAIT cells in renal tissue constitute a tissue‐resident (CD69^+^CD103^+/‐)^ population

**Figure 1:**
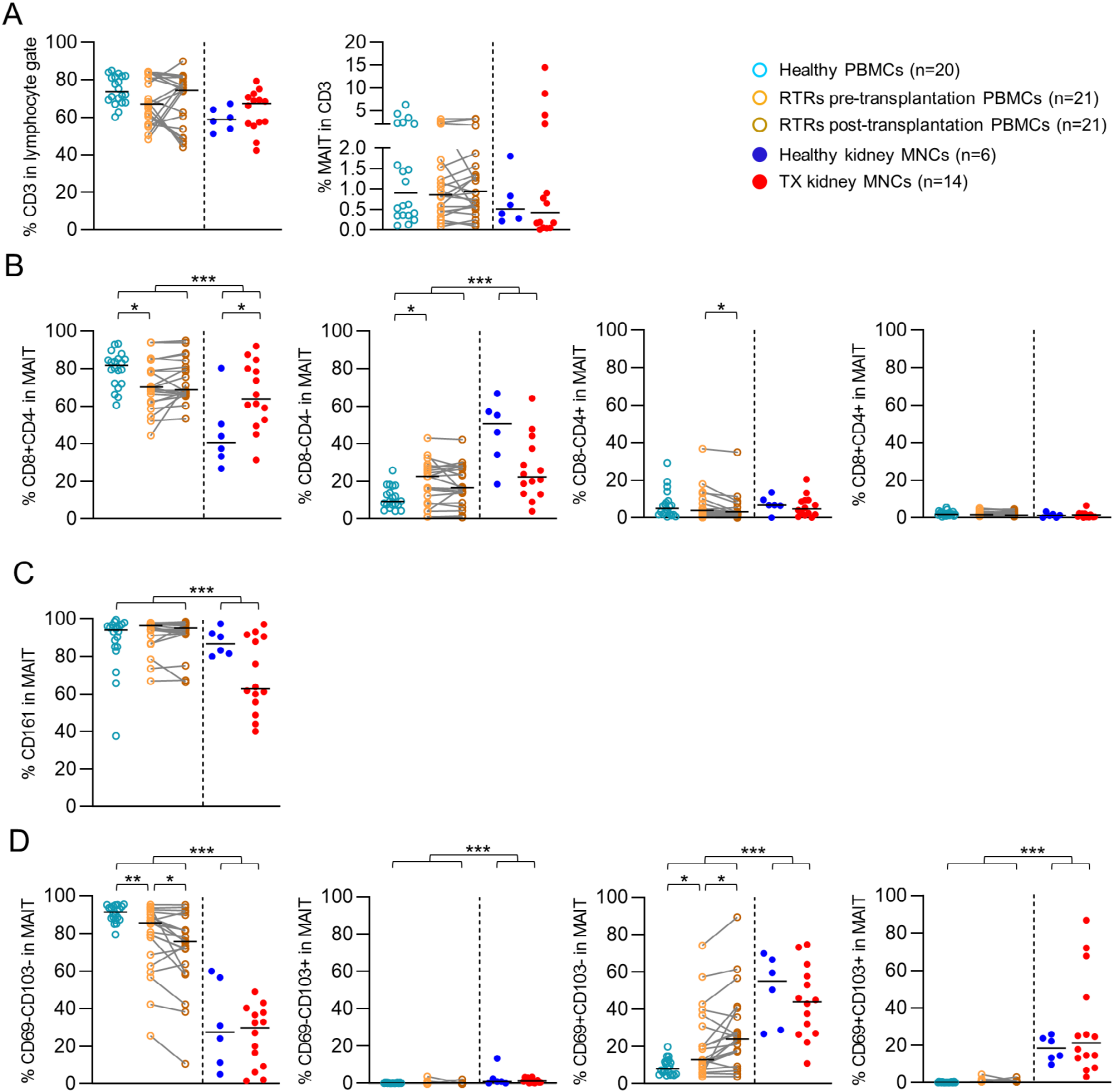
MAIT cells in renal tissue constitute an equal share of the T‐cell population compared with those in circulation and display a tissue‐resident phenotype. (A) Scatterplots of the percentage of CD3^+^ cells in the lymphocyte gate and the percentage of MAIT cells within the CD3 population and scatterplots of the expression of (B) CD4 and CD8, (C) CD161, and (D) CD69 and/or CD103 on MAIT cells in healthy PBMCs, RTRs pretransplantation PBMCs, paired PBMCs post‐transplantation, healthy kidney MNCs, and TX kidney MNCs. The following statistical comparisons were made: kidney MNCs (both healthy and TX) versus PBMCs (healthy and RTRs post‐transplantation) (Mann Whitney U‐test); healthy kidney versus TX kidney MNCs (Mann Whitney U‐test); RTRs pretransplantation versus healthy PBMCs (Mann Whitney U‐test); RTRs pre‐ versus post‐transplantation PBMCs (Wilcoxon signed rank test). The horizontal dash represents the median. Only significant *p*‐values are displayed: *p < 0.05, **p ≤ 0.01, ***p ≤ 0.001. Data shown represent nine flow cytometry experiments with *n* = 2, 4, 9, 2, 21, 10, 16, 15, and 12 donors. Data from 82 unique individuals are shown. RTRs: renal transplant recipients; PBMCs: peripheral blood mononuclear cells; MNCs: mononuclear cells; TX: transplant.

First, we wanted to determine whether MAIT cells in renal tissue comprise an equal share of the T‐cell population compared to the circulation. The percentage of MAIT cells within the T‐cell population appeared to be similar in renal tissue compared to the circulation (respectively 0.51% [0.01‐14.47%] vs. 0.94% [0.11‐6.30%], *p* > 0.05, Fig. 1A). Also, no differences were detected between the transplant versus the healthy kidneys, the pretransplantation versus the healthy blood samples and the pre‐ versus post‐transplantation blood samples (Fig. 1A, Supporting information Fig. S4 and S5). The MAIT cell population in both renal tissue and blood consists of predominantly CD8^+^CD4^−^ (CD8+) and CD8^−^CD4^−^ (double‐negative, DN) MAIT cells with a much smaller population of CD8^+^CD4^+^ and CD8^−^CD4^+^ MAIT cells. However, the distribution of these subsets differed according to anatomical compartment. The proportion of DN MAIT cells was higher in renal tissue than in circulation (27.2% [4.03‐66.7%] vs. 13.9% [0.83‐42.2%], *p* < 0.001), with a significant decrease in the proportion of CD8^+^ MAIT cells (59.9% [26.7‐92.0%] vs. 79.0% [53.8‐95.5%], *p* = 0.001) (Fig. 1B). However, the proportion of CD8^+^ MAITs was higher in the transplant compared to the healthy kidney samples (63.7% [31.3‐92.0% vs. 40.7% [26.7‐80.3%], *p* = 0.04) (Fig. 1B). Interestingly, the proportion of circulating DN MAITs cells was significantly greater in the pretransplantation samples compared to the healthy controls (22.5% [1.13‐43.3%] versus 9.31% [4.13‐25.8%], *p* = 0.032), combined with a lower proportion of CD8^+^ MAIT cells (70.3% [44.7‐94.5%] vs. 81.7% [60.6‐93.3%], *p* = 0.04). Comparison of the pre‐ and post‐transplant blood samples showed no significant differences in the proportion of CD8^+^ and DN MAIT cell populations, except for a significant increase in the small CD8^−^CD4^+^ population (Fig. 1B).

Next, we investigated the expression of CD161, a C‐type lectin used to identify MAIT cells prior to the use of the MR1 tetramer [28]. While the vast majority of MAIT cells expressed this cell surface molecule, the percentage of CD161^+^ MAIT cells was lower in renal tissue than in circulation (80.8% [40.0‐97.4%] vs. 95.5% [40.3‐99.5%, *p* < 0.001) (Fig. 1C). No other differences were observed. We next examined the proportion of MAIT cells displaying a tissue‐resident phenotype, as defined by CD69 and/or CD103 expression. While CD69 is ubiquitously expressed on T cells early after activation, tissue‐resident cells constitutively maintain CD69 expression even under steady state conditions [29]. A substantial proportion of the kidney‐derived MAIT cell population expressed CD69^+^ CD103^+/‐^ and no difference was observed between the transplant and healthy samples (Fig. 1D). There were four kidneys with a relative high amount (>45%) of CD69^+^ CD103^+^ MAIT cells. Interestingly, these four patients had all suffered from recurrent UTI.

As expected, in peripheral blood samples, the percentage of CD103^+^ MAIT cells was negligible in all subjects (Fig. 1D). CD69 was expressed by circulating MAIT cells and the percentage of MAIT cells expressing CD69 was significantly higher in RTRs, both pretransplantation compared to healthy controls and also post‐transplantation when compared to pretransplantation (Fig. 1D).

In summary, the MAIT cells present in renal tissue represent an equal part of the total T‐cell pool found in human kidneys and circulating blood. Renal MAIT cells are more likely to display the DN phenotype and a substantial proportion express CD69, with or without coexpression of CD103, characteristic of the profile associated with T‐cell tissue residency.

### MAIT cells in renal tissue differ phenotypically from circulating MAIT cells

**Figure 2:**
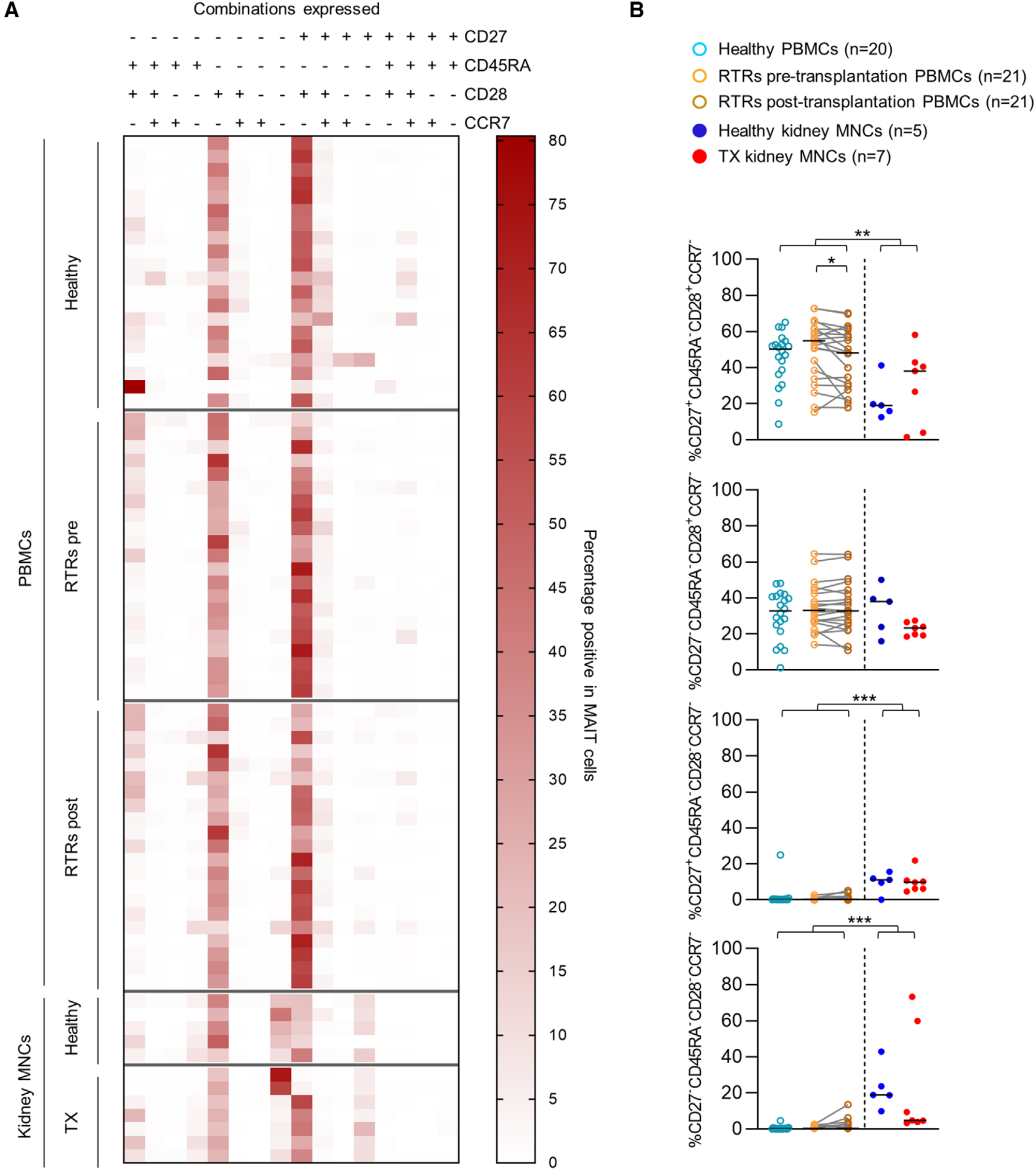
Most MAIT cells display a CD45RA^−^CCR7^−^CD28^+^ expression pattern with or without coexpression of CD27. (A) Heatmap of the expression of CD27/CD45RA/CD28/CCR7 on MAIT cells from healthy PBMCs, RTRs pretransplantation PBMCs, paired PBMCs post‐transplantation, healthy kidney MNCs, and TX kidney MNCs. (B) Scatterplots and statistical analysis of expression patterns with a median abundance of >5 % in at least one of the study groups from heatmap (A). The following statistical comparisons were made: kidney MNCs (both healthy and TX) versus PBMCs (healthy and RTRs post‐transplantation) (Mann Whitney U‐test); healthy kidney versus TX kidney MNCs (Mann Whitney U‐test); RTRs pretransplantation versus healthy PBMCs (Mann Whitney U‐test); RTRs pre‐ versus post‐transplantation PBMCs (Wilcoxon signed rank test). The horizontal dash represents the median. Only significant *p*‐values are displayed: *p < 0.05, **p ≤ 0.01, ***p ≤ 0.001. Data shown represent five flow cytometry experiments with *n* = 21, 10, 16, 15 and 12 donors. Data from 74 unique individuals are shown. RTRs: renal transplant recipients; PBMC: peripheral blood mononuclear cells; MNC: mononuclear cells; TX: transplant.

Circulating MAIT cells generally express CD27 and CD28 [30]. To determine whether this was also the case for MAIT cells in the kidney, we evaluated the expression of these markers in addition to CCR7 and CD45RA. This combination of markers, in the general CD8^+^ T‐cell pool, is indicative of T‐cell differentiation [31, 32, 33, 34]. MAIT cells in the kidney and the circulation predominantly displayed a CD45RA^−^CCR7^−^CD28^+^ phenotype with or without CD27 coexpression (Fig. 2A). Interestingly, in renal tissue, the percentage of MAIT cells with a CD27^+^CD45RA^−^CCR7^−^CD28^+^ phenotype was lower than the circulating population, while there was a significant increase in the proportion of MAIT cells displaying a CD45RA^−^CCR7^−^CD28^−^ phenotype, either with or without coexpressing CD27 (Fig. 2B). Thus, the proportion of MAIT cells that had lost their expression of CD28 with or without the simultaneous loss of CD27 was higher in the kidney than in circulation. There were no differences between the transplants and healthy kidney samples, while in circulation, there was a marked decline in cells with a CD27^+^CD45RA^−^CCR7^−^CD28^+^ phenotype in the post‐ compared to pretransplantation samples (Fig. 2B).

**Figure 3:**
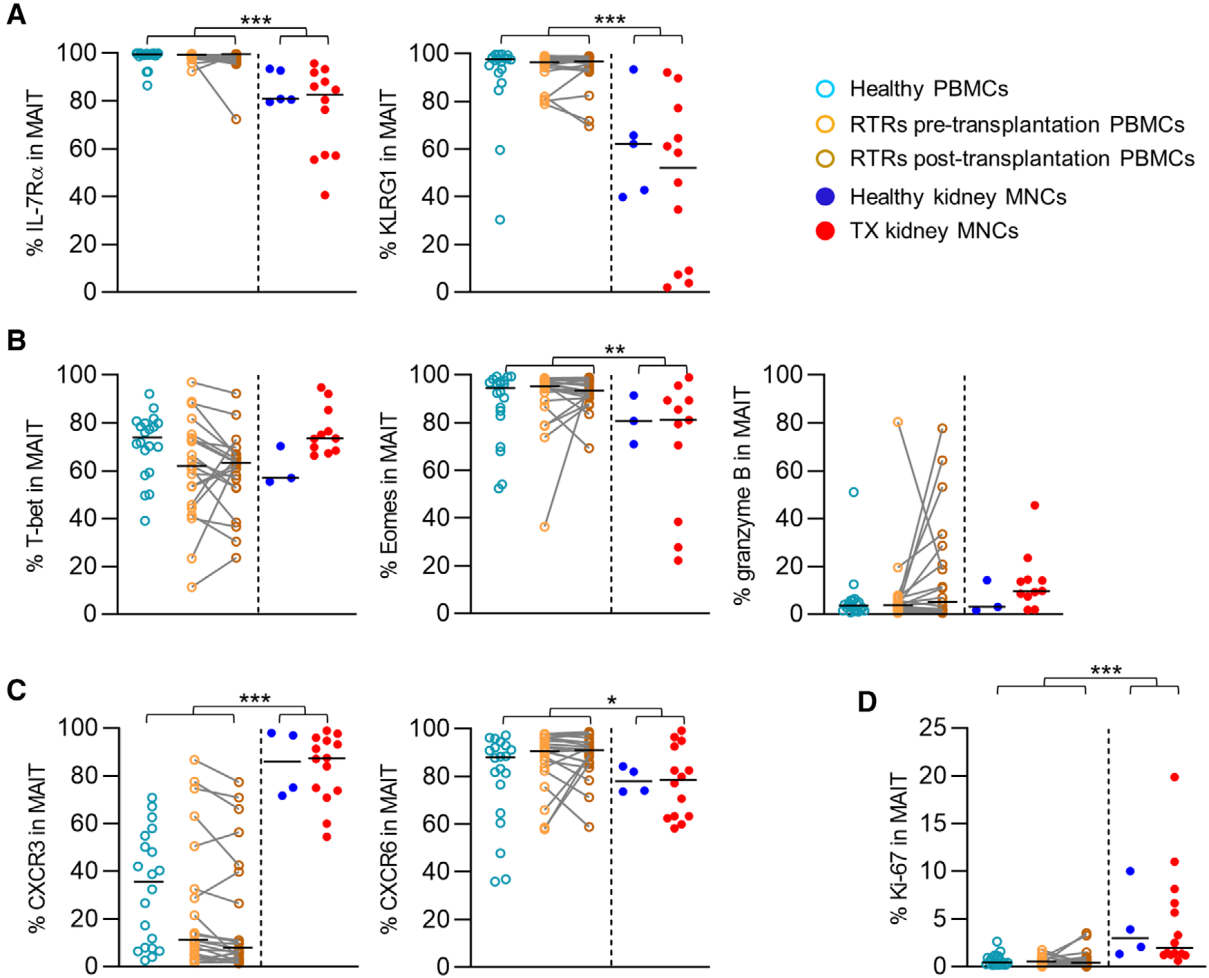
MAIT cells in renal tissue phenotypically differ from circulating MAIT cells. Scatterplots of the percentage of MAIT cells expressing of (A) IL‐7Rα and KLRG1 (B) T‐bet, eomes, granzyme B, and ki‐67 (C) CXCR3 and CXCR6 in healthy PBMCs, RTRs pretransplantation PBMCs, paired PBMCs post‐transplantation, healthy kidney MNCs, and TX kidney MNCs. The following statistical comparisons were made: kidney MNCs (both healthy and TX) versus PBMCs (healthy and RTRs post‐transplantation) (Mann Whitney U‐test); healthy kidney versus TX kidney MNCs (Mann Whitney U‐test); RTRs pretransplantation versus healthy PBMCs (Mann Whitney U‐test); RTRs pre‐ versus post‐transplantation PBMCs (Wilcoxon signed rank test). The horizontal dash represents the median. Only significant *p*‐values are displayed: *p < 0.05, **p ≤ 0.01, ***p ≤ 0.001. Data shown represent nine flow cytometry experiments with *n* = 2, 4, 9, 2, 21, 10, 16, 15, and 12 individuals. (A) A total of 79 unique individuals are shown (healthy PBMC = 20, RTRs pre‐/post‐ transplantation = 21, healthy kidney = 5, and TX kidney = 12). (B) A total of 76 unique individuals are shown (healthy PBMC = 20, RTRs pre‐/post‐transplantation = 21, healthy kidney = 3, and TX kidney = 11). (C) A total of 80 unique individuals are shown (healthy PBMCs = 20, RTRs pre‐/post‐transplantation = 21, healthy kidney = 4, and TX kidney = 14). RTRs: renal transplant recipients; PBMCs: peripheral blood mononuclear cells; MNCs: mononuclear cells; TX: transplant.

Since in the overall CD8+ T‐cell pool, loss of CD28 generally associated with a cytotoxic phenotype [34], we wondered whether this would also be true for MAIT cells in the kidney. Therefore, we evaluated the expression of markers that normally fit distinct functional profiles of αβ T‐cells, such as interleukin‐7 receptor α‐chain (IL‐7Rα), which is frequently expressed on CD27^+^CD28^+^ CD8 T cells and is lost during differentiation toward a more cytotoxic memory profile [31], and killer cell lectin‐like receptor subfamily G member 1 (KLRG1), which is an inhibitory TCR specific to differentiated cytotoxic T cells [35, 36]. In renal tissue, the percentage of MAIT cells expressing IL‐7Rα was lower compared to in circulation (80.9% [40.6‐95.7%] vs.99.6% [72.6‐100.0%], *p* < 0.001). However, the percentage of MAIT cells expressing KLRG1 was also lower in the kidney (58.6% [2.10‐93.4%] vs. 97.2% [30.4‐99.7%], *p* ≤ 0.001, Fig. 3A). There were no differences noted in the expression of these markers between the healthy and transplant kidney samples or in circulation between the pretransplantation RTRs (upon sample collection ESRD) and healthy controls or between the RTRs pre‐ and post‐transplantation. Thus, no effects of ESRD or transplantation were found in either the renal or circulating cell populations.

Next, we compared the expression of T‐box transcription factor (T‐bet) and eomesdermosin (Eomes), which regulate the expression of several molecules associated with cytotoxic cellular phenotypes (e.g., serine protease granzyme B) [32, 34]. A substantial proportion of the MAIT cell population in the kidney expressed T‐bet, which was not different when compared to the circulating population (Fig. 3B). However, the proportion of MAIT cells expressing Eomes was significantly lower in renal tissue than in the circulating population (81.0% [22.2‐99.0%] vs. 93.5% [52.5‐99.4%], *p* = 0.002, Fig. 3B). In contrast to what would be expected from a larger CD28‐negative population and substantial T‐bet expression, granzyme B was only expressed in a small subpopulation of renal MAIT cells, which was similar in size to the subpopulation observed in circulation (Fig. 3B). Neither ESRD nor transplantation affected the expression of these markers in MAIT cells regardless of localization.

Next, we evaluated the expression of chemokine receptors CXCR3 and CXCR6, which allow cells to migrate to distinct anatomic compartments such as the kidney [25, 37–41]. The percentage of MAIT cells expressing CXCR6 was lower in renal tissue compared with the circulating population (78.6% [58.3‐99.2%] vs. 89.1% [36.0‐98.5%], *p* = 0.045, and 46.7% [3.97‐93.4%] vs. 73.6% [14.2‐99.2%], *p* = 0.03, respectively), while the percentage of MAIT cells expressing CXCR3 was higher in the kidney (87.3% [54.5%‐99.0%] vs. 11.8% [1.82%‐77.7%], *p* < 0.001) (Fig. 3C). Again, the expression of these markers was not affected by ESRD or transplantation. Finally, we investigated expression of Ki‐67, a marker of actively dividing cells [42]. Ki‐67 was expressed by a low but significantly higher number of MAIT cells in renal tissue compared to the circulating population, reflective of the presence of more actively cycling MAIT cells in the kidney (2.27% [0.63‐19.9%] vs. 0.43% [0.13‐3.61], *p* < 0.001, Fig. 3D). The expression of Ki‐67 in MAIT cells was not affected by ESRD or transplantation in either compartment.

In summary, MAIT cells predominantly expressed a CD27^+/−^CD45RA^−^CCR7^−^CD28^+^IL‐7Rα^+^KLRG1^+^ profile. However, a distinct CD27^+/−^CD45RA^−^CCR7^−^CD28^−^ subpopulation was detected in the kidney that was virtually absent in circulation. Relative to their circulating counterparts, kidney‐derived MAIT cells were less likely to express IL‐7Rα, KLRG1, and CXCR6, and more likely to express CXCR3 and Ki‐67. This expression pattern was not affected by ESRD or renal transplantation in either compartment.

### Tissue‐resident MAIT cells display a distinct phenotype

**Figure 4:**
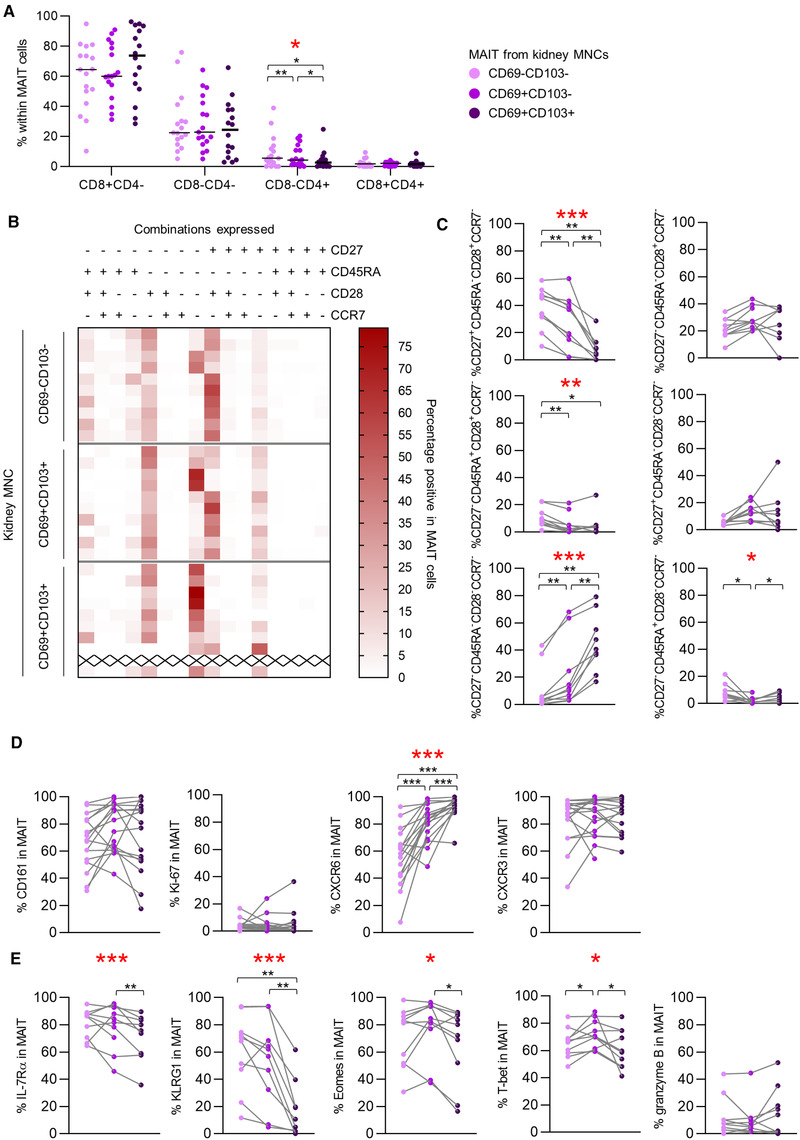
Tissue‐resident MAIT cells display a distinct phenotype. (A) Scatterplot of the expression of CD4 and/or CD8 on CD69‐ and CD103‐expression‐defined MAIT cell populations from kidney MNCs. The horizontal dash represents the median. (B) Heatmap of the CD27/CD45RA/CD28/CCR7 expression pattern of CD69^–^CD103^−^, CD69^+^CD103^−^, and CD69^+^CD103^+^ MAIT cell populations from kidney MNCs. (C) Scatterplots of expression patterns with a median abundance of >5 % in at least one of the study groups from heatmap. (B) Lines connect measurements within one individual. Scatterplots of the percentage of cells expressing (D) CD161, Ki‐67, CXCR6, and CXCR3 (E) IL‐7Rα, KLRG1, eomes, T‐bet, and granzyme B among CD69^–^CD103^−^, CD69^+^CD103^−^, and CD69^+^CD103^+^ MAIT cell populations from kidney MNCs. Lines connect measurements within one individual. (A,C,D,E) A statistical comparison was made to evaluate differences between the three (CD69^–^CD103^−^, CD69^+^CD103^−^, and CD69^+^CD103^+^) populations (Friedman). In case of a significant difference (indicated by the * in red), a post‐hoc Wilcoxon signed rank test was performed. Only significant *p*‐values are displayed: *p < 0.05, **p ≤ 0.01, ***p ≤ 0.001. A and D: Data shown represent five flow cytometry experiments with *n* = 2, 4, 9, 2, and 12 individuals. A total of 17 unique individuals are shown (healthy kidney = 4 and TX kidney = 13) (one sample did not contain sufficient CD69^+^CD103^+^ MAIT cells for analysis). B, C, and E: data shown represent one experiment with *n* = 10 unique individuals (healthy kidney = 3 and TX kidney = 7) (one sample did not contain sufficient CD69^+^CD103^+^ MAIT cells for analysis (represented by x in B). PBMCs: peripheral blood mononuclear cells; MNCs: mononuclear cells; TX: transplant.

Analysis of the tissue‐resident populations revealed that, among CD69^+^CD103^+/‐^ MAIT cells, the proportions of the CD8^+^ and DN populations were similar to the nontissue resident CD69^−^CD103^−^ MAIT population. Even though the proportion of CD8^−^CD4^+^ MAIT cells was generally small, expression of CD69^+^CD103^+/‐^ was associated with relatively fewer CD8^−^CD4^+^ MAIT cells compared to CD69^−^CD103^−^ MAIT cells (Fig. 4A). CD69^+^CD103^+/‐^ MAIT cells predominantly displayed the CD27^−^CD45RA^−^CCR7^−^CD28^−^ profile and only a few CD69^+^CD103^+/‐^ MAIT cells expressed the costimulatory molecules CD27 and CD28 (Fig. 4B and 4C).

The core signature of human tissue‐resident T‐cells generally includes low expression of KLRG‐1 and/or Eomes, low/intermediate amounts of T‐bet and high levels of CXCR6 [43, 44, 45, 46].

Also, in our analysis, the percentage of cells expressing CXCR6 was higher among CD69^+^CD103^+/‐^ populations (Fig. 4D). The percentage of MAIT cells expressing KLRG1 was indeed lower among renal‐derived CD69^+^CD103^+^ cells compared to either CD69^+^CD103^−^ or CD69^−^CD103^−^ MAIT cells, yet there was no significant difference when comparing the CD69^+^CD103^−^ and CD69^−^CD103^−^ populations (Fig. 4E). For Eomes, and also for IL‐7Rα, only expression of CD103 correlated with a decreased percentage of MAIT cells expressing these markers (Fig. 4E). In contrast to what is generally accepted for tissue‐resident T‐cells, the percentage of cells expressing T‐bet was higher among CD69^+^CD103^−^ cells than among CD69^−^CD103^−^ MAIT cells while, in the CD69^+^CD103^+^ population, the percentage of cells expressing T‐bet was lower than in the CD69^+^CD103^−^ population (Fig. 4E). No significant differences were found regarding the percentage of cells expressing CD161, CXCR3, granzyme B, or Ki‐67 (Fig. 4D and 4E).

In summary, CD69^+^CD103^+/‐^ MAIT cells predominantly displayed a CD27^−^CD45RA^−^CCR7^−^CD28^−^ phenotype. CD69^+^CD103^+/‐^ MAIT cells more often expressed CXCR6 than CD69^−^CD103^−^ MAIT cells, while IL‐7Rα, KLRG‐1, and Eomes were less frequently expressed.

### MAIT cells in renal tissue are more often polyfunctional than circulating MAIT cells

**Figure 5:**
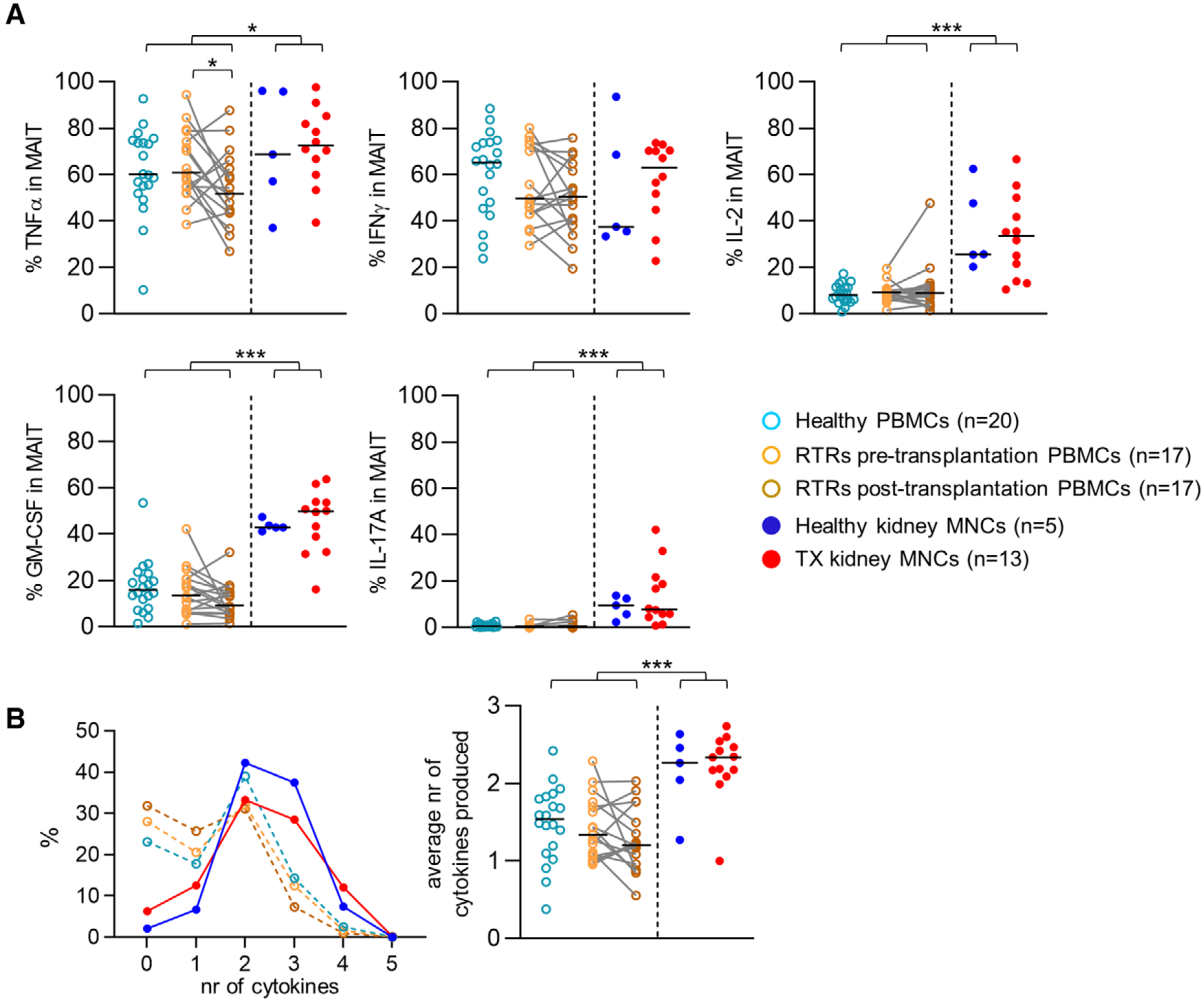
MAIT cells in renal tissue are more often polyfunctional after stimulation with PMA‐ionomycin than circulating MAIT cells. (A) Scatterplots of the percentage of TNFα‐, IFNү‐, IL‐2‐, GM‐CSF‐, or IL‐17A‐producing MAIT cells from healthy PBMCs, RTRs pretransplantation PBMCs, paired PBMCs post‐transplantation, healthy kidney MNCs, and TX kidney MNCs after stimulation with PMA and Ionomycin. (B) Graph of the median percentage of MAIT cells (*Y*‐axis) producing the stated number of cytokines (*X*‐axis) and scatterplot of the average number of cytokines produced by MAIT cells from healthy PBMCs, RTRs pretransplantation PBMCs, paired PBMCs post‐transplantation, healthy kidney MNCs and TX kidney MNCs after stimulation with PMA and Ionomycin. The following statistical comparisons were made: kidney MNCs (both healthy and TX) versus PBMCs (healthy and RTRs post‐transplantation) (Mann Whitney U‐test); healthy kidney versus TX kidney MNCs (Mann Whitney U‐test); RTRs pretransplantation versus healthy PBMCs (Mann Whitney U‐test); RTRs pre‐ versus post‐transplantation PBMCs (Wilcoxon signed rank test). The horizontal dash represents the median. Only significant *p*‐values are displayed: *p < 0.05, **p ≤ 0.01, ***p ≤ 0.001. RTRs: renal transplant recipients; PBMCs: peripheral blood mononuclear cells; MNCs: mononuclear cells, TX: transplant. Data shown are representative of eight independent flow cytometry experiments with *n* = 3, 8, 9, 10, 12, 12, 7, and 12 individuals per experiment. A total of 72 unique individuals are shown.

Next, we evaluated the functional profile of MAIT cells by stimulating them with PMA‐ionomycin. After stimulation, the percentage of MAIT cells producing TNFα, IL‐2, granulocyte‐macrophage colony‐stimulating factor (GM‐CSF), or interleukin 17A (IL‐17A) was significantly higher in renal tissue compared to the circulation (Fig. 5A). A substantial proportion of the MAIT cell population produced interferon γ (IFNү), which did not differ between MAIT cells in the kidney and those in circulation. MAIT cells were also significantly more often polyfunctional in renal tissue than in circulation, with a higher average number of different cytokines produced by each MAIT cell compared to the circulating population (Fig. 5B). TNFα and IFNү were often produced simultaneously with or without the concomitant production of GM‐CSF. The functional combinations of cytokines produced by MAIT cells also differed between the kidney and the circulation (Supporting information Fig. S6). The percentage of MAIT cells that produced TNFα, IFNү, IL‐2, GM‐CSF, or IL‐17A and the average number of cytokines produced did not vary significantly between healthy and transplanted kidneys or between healthy and pretransplantation blood samples. In contrast, the percentage of circulating MAIT cells producing TNFα was higher prerelative to post‐transplantation, while there was no difference between the pre‐ and post‐transplantation samples in the proportion of cells producing IFNү, IL‐2, GM‐CSF, or IL‐17A or in the average number of functions (Fig. 5A and 5B).

In summary, after stimulation with PMA‐ionomycin, the percentage of MAIT cells producing TNFα, IL‐2, GM‐CSF, or IL‐17 was higher in renal tissue than in circulation and the former were also more often polyfunctional.

### CD103+ MAIT cells favor an IL‐2, GM‐CSF, and IL‐17‐producing profile

**Figure 6:**
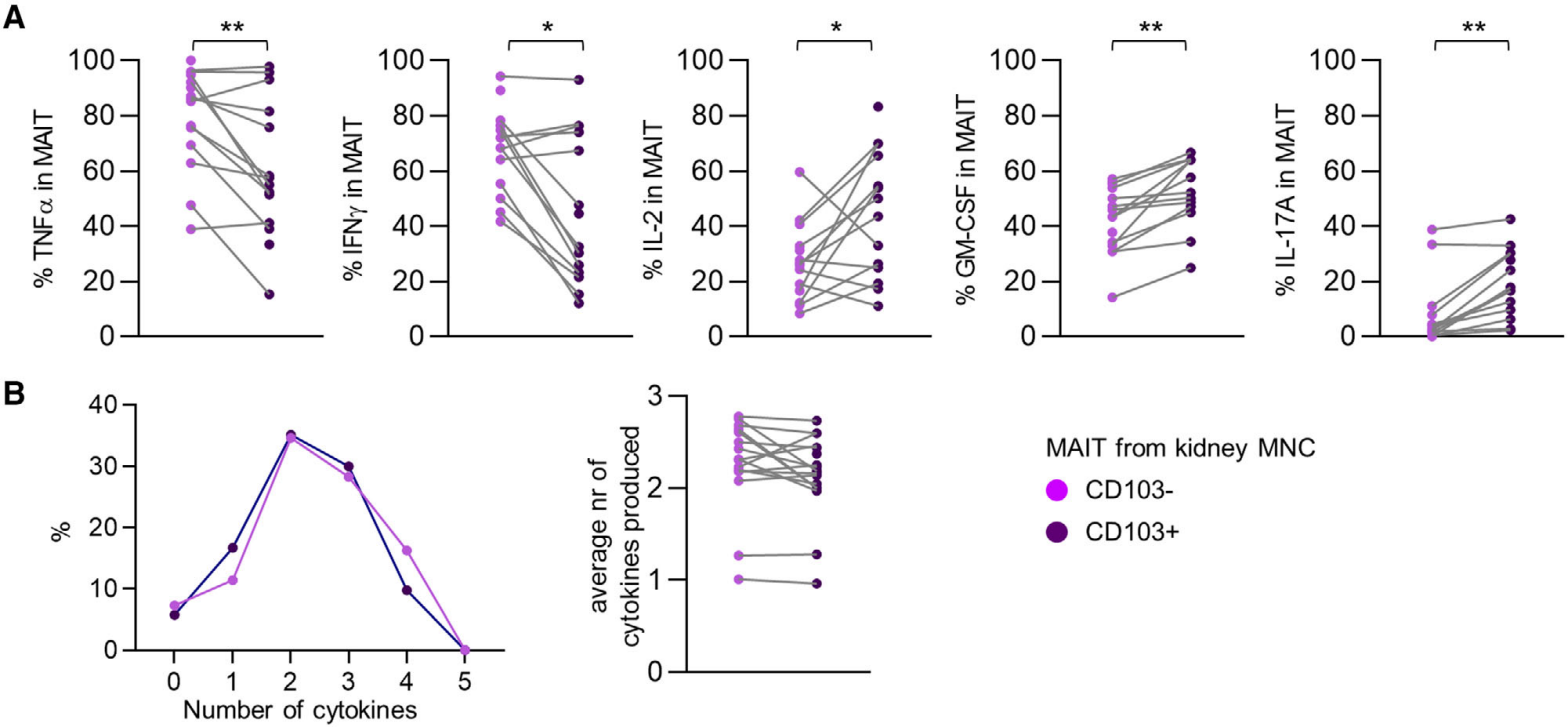
CD103^+^ MAIT cells favor an IL‐2, GM‐CSF, and IL‐17‐producing profile. (A) Scatterplot of the percentage of TNFα‐, IFNү‐, IL‐2‐, GM‐CSF‐, or IL‐17A‐producing MAIT cells within CD103‐negative and CD103‐positive MAIT cell populations from kidney MNCs after stimulation with PMA and Ionomycin. (B) Graph of the median percentage of MAIT cells (*Y*‐axis) producing the stated number of cytokines (*X*‐axis) and scatterplot of the average number of cytokines produced by CD103‐negative and CD103‐positive MAIT cells from kidney MNCs after stimulation with PMA and Ionomycin. Wilcoxon signed rank test was used for statistical comparison. Only significant *p*‐values are displayed: *p < 0.05, **p ≤ 0.01, ***p ≤ 0.001. Data shown are representative of two independent flow cytometry experiments with *n* = 7 and 12 individuals per experiment. A total of 16 unique individuals are shown (healthy kidney = 3 and TX kidney = 13) (two samples did not contain sufficient CD103+ MAIT cells for analysis and one sample did not contain sufficient CD103‐ MAIT cells for analysis).

Since CD69 is also upregulated during immune activation [47], only CD103 was used to identify tissue‐resident MAIT cells. A comparison of CD103+ cells to CD103‐ cells revealed that the proportion of CD103^+^ MAIT cells producing TNFα or IFNү poststimulation was lower compared to CD103^−^ MAIT cells. Instead, the percentage of CD103^+^ MAIT cells producing IL‐2, GM‐CSF, or IL‐17A was higher compared to their CD103^−^ counterpart (Fig. 6A). There were no significant differences in the average number of cytokines produced (Fig. 6B), yet the combinations of cytokines produced differed significantly (Supporting Information Fig. S7A and S7B).

In summary, CD103^+^ MAIT cells in the kidney preferentially adopted an IL‐2, GM‐CSF, and IL‐17A‐producing fate.

**Table 1:** Demographics. Characteristics of the participants in this study

	Healthy kidneys	Kidney transplants	Healthy donors	RTRs pretransplantation	RTRs post‐transplantation
	n = 6	n = 14	n = 20	n = 21	n = 21
Sex, men (%)	50%	67%	55%	71%	71%
Age in years, median [range]	70.4 [57–78]	48 [22–71]	55.8 [37–71]	60 [30–74]	60 [30–74]
Diabetes mellitus (%)	Unknown	28.6%	0%	38.1%	47.6%
Original renal disease of the allograft recipient (%)	N/A		N/A		
‐FSGS		21.4%		4.8%	
‐Renovascular		21.4%		71.5%	
‐SLE		14.3%		–	
‐IgAN		7.1%		4.8%	
‐ADPKD		14.1%		4.8%	
‐Polyarthritis nodosa		7.1%		–	
‐Congenital anatomical abnormality		7.1%		4.8%	
‐Idiopathic membranous glomerulopathy		7.1%		–	
‐Alport syndrome		–		4.8%	
‐Unknown		–		4.8%	
Cause of transplant failure as reported in the medical file (%)	N/A		N/A	N/A	N/A
‐RUTI		35.7%			
‐Rejection		42.8%			
‐ATN		14.3%			
‐BK		7.1%			
Months since renal transplantation, median [range]	N/A	48 [0.3‐120]	N/A	N/A	11.0 [8.0‐14.0]
Number of immunosuppressive agents at time of sample collection, median [range]	N/A	1.5 [1‐4]	N/A	N/A	3 [2‐3]

ADPKD, autosomal dominant polycystic kidney disease; ATN, acute tubular necrosis; BK, polyomavirus BK‐induced nephropathy; FSGS, focal segmental glomerulosclerosis; IgAN, IgA nephropathy; N/A, not applicable; RTRs, renal transplant recipients; RUTI: recurrent urinary tract infection; SLE: systemic lupus erythematosus.

## Discussion

Using the MR1 tetramer, which enables distinguishing MAIT cell populations based on TCR specificity, we evaluated the phenotype and function of MAIT cells in renal tissue relative to their circulating counterparts. Kidney‐derived MAIT cells represent a distinct, tissue‐resident population that, as expected, differed from the MAIT cells found in circulation. First, in the kidney, the proportion of DN MAIT cells was higher than in circulation. Also, relatively more DN MAIT cells were seen in circulation before and after transplantation. Previous studies have pointed out that MR1‐dependent stimulation, similar to our results after nonspecific stimulation with PMA‐ionomycin, results in the downregulation of the CD8‐receptor and that DN MAIT cells in fetal tissues are more mature than CD8^+^ MAIT cells [48]. Therefore, it has been suggested that DN MAIT cells are likely derived from the CD8^+^ MAIT cell pool and more developmentally mature.

MAIT cells expressed CD161 less frequently in the kidney compared to in circulation. CD161 is downregulated following antigen stimulation [49], similar to IL‐7Rα, which was also less frequently expressed by MAIT cells in the kidney. Taken together, these data suggest that MAIT cells in the kidney were recently exposed to their cognate antigen. The percentage of cells expressing CD28, a costimulatory molecule lost upon differentiation into the cytotoxic fate [34], was also lower among MAIT cells from the kidney. However, this did not correspond to an increased proportion of cells expressing the cytotoxic marker granzyme B. The percentage of MAIT cells expressing KLRG1 was lower in the kidney than in circulation but, since TGF‐β downregulates KLRG1, its ability to serve as a marker of T‐cell differentiation in tissues expressing high levels of TGF‐β has been questioned [36].

A more in‐depth analysis of the CD69^+^CD103^+/‐^ populations revealed that these cells differed from CD69^−^CD103^−^ MAIT cells. CD69^−^CD103^−^ MAIT cells in the kidney largely resembled the circulating population, while CD69^+^CD103^+/‐^ MAIT cells appeared to constitute a distinct population with characteristics consistent with other tissue‐resident memory T‐cell populations, such as high CXCR3, and CXCR6 expression levels and low IL‐7Rα, KLRG‐1, and Eomes expression levels [43–45, 50]. CXCR6 was also expressed by MAIT cells in peripheral blood but less frequently among the CD69^−^CD103^−^ MAIT cells in the kidney. This pattern of CXCR6 expression was also observed in brain CD69^+^CD103^+/‐^ and CD69^−^CD103^−^ T‐cells [44]. Chemokine receptors, such as CXCR6, are involved in trafficking and positioning T‐cells within tissues using chemokine gradients and adhesion molecules [51]. The downregulation of this receptor likely follows engaging its respective chemokine target and it is possible that only cells lacking CXCR6 can egress into circulation.

The frequency of MAIT cells that secrete IL‐2, GM‐CSF, or IL‐17A in response to PMA‐ionomycin stimulation was generally low in circulation and comparatively high in renal tissue; furthermore, the latter more often produced combinations of different cytokines and, therefore, are more polyfunctional. CD69 could no longer be used as a reliable marker of tissue residency in these experiments since its expression can be upregulated by immune activation. Nevertheless, distinguishing MAIT cell populations according to CD103 expression revealed that CD103^+^ MAIT cells favor an IL‐2, GM‐CSF, and IL‐17A‐producing profile. These cytokines are produced by other tissue‐resident T‐cells [43, 44] and while data on the function of tissue‐resident MAIT cells are scarce, those in the human oral mucosa also produce IL‐17 [52]. Furthermore, MAIT cells residing in the human female genital tract are also biased toward IL‐17 (and IL‐22) production [4]. In these studies, production of IL‐2 and GM‐CSF was not evaluated. IL‐2, GM‐CSF, and IL‐17A stimulate the proliferation of lymphocytes and the recruitment of monocytes and neutrophils [53, 54, 55, 56]; their rapid production in response to immune insult illustrates the functional diversification of tissue‐resident MAIT cells from their circulating counterparts.

These features suggest that MAIT cells in the kidney serve as the first line of defense against ascending uropathogens. Local protection provided by tissue‐resident T‐cells against invading pathogens has been demonstrated in a variety of other barrier tissues [57, 58]. In the current study, the kidneys from four participants contained a substantially higher proportion of CD69^+^CD103^+^ MAIT cells. In these four participants, the cause of transplant failure reported by their nephrologists was recurrent UTIs, suggesting that this infection contributes to the development or persistence of the CD69^+^CD103^+^ MAIT cell population in the kidney.

In circulation, the number of MAIT cells was not decreased pre‐ or 12‐months post‐transplantation. This is in contrast with studies that describe fewer circulating MAIT cells in patients on dialysis and also after transplantation compared to healthy controls [24, 25]. This may be explained by methodological differences, since, in one study [24], MAIT cells were not identified based on TCR specificity but by the expression of CD3, CD161, and TCR Vα7.2 chain, and, in the other study [25], only CD8^+^ circulating MAIT cells were analyzed. In both the latter and the present study, the activation state of circulating MAIT cells, assessed by CD69 expression [25] was elevated, both pre‐ and post‐transplantation. This may be a consequence of more frequent infections in these patients. Furthermore, post‐transplantation, we found that a decreased percentage of circulating MAIT cells was producing TNFα, potentially due to the use of immunosuppressive agents [59]. Nevertheless, in the current study, there was no evidence to suggest that a decreased number or the impaired function of circulating MAIT cells are responsible for the frequent infections observed in RTRs both before and after transplantation. Neither did we find a difference in the abundance of MAIT cells in the transplant versus the healthy kidneys. In this prospect, it should be noted that we were only able to evaluate the percentage of MAIT cells within the T‐cell population and not absolute MAIT cell numbers. Furthermore, the percentage of MAIT cells could also be diluted by the influx of alloreactive T‐cells. Most transplants were removed after the immunosuppression was already tapered down to a minimum. Therefore, the lack of difference between the amount of MAIT cells between transplant versus healthy kidneys should be interpreted with caution. In addition to this, it is currently unclear whether MAIT cells may also play a role in nonmicrobiological immune‐mediated pathology in the kidney; it has been suggested that MAIT cells contribute to the fibrotic process of CKD [5].

In summary, circulating MAIT cells collected from RTRs before and after transplantation were not decreased in number but assumed a DN phenotype more often than those from healthy controls in addition to showing signs of recent activation. The MAIT cells present in human renal tissue represent a distinct tissue‐resident cell population with a CD27^−^CD45RA^−^CCR7^−^CD28^−^ phenotype and a capacity to produce IL‐2, GM‐CSF, and IL‐17A that is more potent than circulating MAIT cells. Taken together, the findings described here suggest that these tissue‐resident MAIT cells serve to recruit monocytes and neutrophils to the kidney upon injury or immune insult and, as such, may contribute to the protection of the kidney against invading microorganisms.

We have shown several differences between MAIT cells in the kidney and MAIT cells from the circulation, which requires further study. Elucidation of the exact functional role of MAIT cells in the kidney might help us to understand the pathogenesis of recurrent UTI, which at this moment remains a significant clinical problem.

## Methods

### Patients and samples

Samples were obtained from the Biobank Renal Diseases of the Amsterdam UMC location AMC. In this Biobank, patient samples, such as blood and renal tissue (residual tissue after transplantectomy), are collected and stored from healthy living kidney donors and RTRs that are followed before and after renal transplantation. This study was conducted according to the principles outlined in the Declarations of Helsinki and Istanbul and all participants provided written informed consent prior to enrollment in the Biobank. Additionally, residual tissue from patients who underwent tumor nephrectomy (renal tissue distant from the tumor) was donated by the Department of Pathology and also stored in the Biobank. These tissues were processed anonymously according to the Federation of Dutch Medical Scientific Societies’ Code of Conduct (Human Tissue and Medical Research: Code of Conduct for Responsible Use, 2011 www.federa.org).

### Peripheral blood mononuclear cells (PBMCs)

Blood samples were obtained once from healthy controls (living kidney donors prior to surgery, n = 20) and twice from RTRs (before and 1‐year after transplantation, n = 21). Characteristics of the participants in this study are displayed in Table 1. PBMCs were isolated from sodium heparin blood by standard density gradient centrifugation and subsequently cryopreserved until the day of analysis.

### Renal tissue

Healthy renal tissue samples (n = 6) were obtained from kidneys that were surgically removed due to renal cell carcinoma (distant nontumorous tissue from the contralateral pole of the kidney) and transplant renal tissue (n = 14) was obtained from explanted renal allografts after transplant failure. These samples are further referred to as healthy and transplant kidney samples, respectively. Slices of kidney cortex were chopped into 1‐mm cubes with the McIlwain Tissue Chopper (Ted Pella, Redding, CA, USA), transferred to 50 mL tubes and washed with cold PBS until no blood was visibly present and the supernatant was clear. Preheated (37°C) digestion medium was added, 40 mL per 10 g of tissue (DNAse I type IV [50 KU/mL] (Sigma Aldrich, Zwijndrecht, Netherlands), collagenase type IV (0.5 mg/mL) (Wortington Biochemical, Lakewood, NJ, USA), BSA (60 mg/mL) (Sigma Aldrich), 20 μL/mL fetal calf serum (FCS, VWR International BV, Amsterdam, Netherlands), TRIS (0.025 M) (Merck BV, Amsterdam, Netherlands), penicillin streptomycin (Biochrom GMBH, Berlin, Germany) in HBSS (Westburg BV, Leusden, Netherlands)), and incubated in a shaker for 20 min at 37°C. The warm suspension was transferred to a C‐tube (Miltenyi, Bergisch Gladbach, Germany) and subjected to the M_spleen_04.01 program on the GentleMacs (Miltenyi). The digestion medium was deactivated with cold PBS and the resulting cell suspension passed through a cell strainer to obtain a single cell suspension, which was subjected to standard density gradient centrifugation according to manufactures protocol (Lymphoprep, Abbott Diagnostics Technologies AS, Oslo Norway). The isolated mononuclear cells (kidney MNCs) were cryopreserved until the day of analysis.

### Cryopreservation

The isolated mononuclear cells (kidney MNCs and PBMCs) were cryopreserved in IMDM supplemented with 20% FCS, 0.00036 v/v% β‐mercaptoethanol and 5% DMSO.

### Flow cytometry

We used the fluorescently‐labeled 5‐OP‐RU MR1‐tetramer (NIH, Bethesda, MD, USA [16]) in conjunction with 16‐color flow cytometry to identify and characterize MAIT cells in PBMC and kidney MNC samples. Measurements were performed on an LSRFortessa flow cytometer (BD Biosciences). For each sample, 2 × 10^6^ PBMCs or 0.5 × 10^6^ to 10 × 10^6^ kidney MNCs were analyzed. The volume of each staining reaction was relative to the number of cells and antibody concentration kept constant. Cells were incubated with the BV421‐labeled human MR1‐tetramer 5‐A‐RU complex or human MR1‐tetramer‐6‐FP complex as the negative control for 30 min at 4°C in the dark, after which the surface antibodies (Supporting information Table S1) were added for 30 min under the same conditions. Dead cells were excluded using the fixable viability dye eFluor780, eFluor455UV, or viability dye eFluor506 (eBioscience Inc., Thermo Fisher Scientific, San Diego, CA, USA). Monoclonal antibodies with intracellular targets (Supporting information Table S1) were added after the fixation and permeabilization of the cells using the FoxP3/Transcription Factor Staining Set (eBioscience Inc.). Published methods for flow cytometry and cell sorting for immunological purposes were followed [60]. The gating strategy used for the phenotypic analysis can be found in Supporting information Fig. S1.

The effect of the digestion method used to isolate mononuclear cells from kidney on expression of markers on MAIT cells was evaluated on freshly isolated PBMCs. The only marker that was affected by the digestion was CCR6. Therefore, CCR6 was excluded from further analyses (Supporting information Fig. S2).

Tissue sample limitations resulted in the exclusion of some samples from some panels. Samples were only analyzed when they contained over 50% live cells within the lymphogate, as assessed by a viability dye, and MAIT cells were only characterized if their total cell count exceeded 15.

### Stimulation assay

MAIT cells were stimulated as previously described [47, 61]. PBMCs and kidney MNCs were thawed in the presence of DNAse I (200 KU/mL), washed, and allowed to recover overnight in untreated, round‐bottom, 96‐well plates (Corning) in culture medium (RPMI supplemented with 10% FCS and penicillin streptomycin) at a concentration of 20 × 10^6^/mL (100 μL/well).

The next morning, phorbol 12‐myristate 13‐acetate (PMA, 10 ng/mL; Sigma Aldrich) and ionomycin (1 μg/mL; Sigma Aldrich) were added to stimulate the cells. Medium alone was added as the negative control. All incubations were performed in culture medium in the presence of αCD28 (clone 15E8; 2 μg/mL), αCD29 (clone TS 2/16; 1 μg/mL), brefeldin A (10 μg/mL, Invitrogen); and GolgiStop (BD Biosciences) in a final volume of 200 μL for 4 h at 37°C and 5% CO_2_.

Subsequently, the cells were incubated for 30 min with PE‐ and BV421‐labeled 5‐OP‐RU‐MR1‐tetramers, after which the surface antibodies (Supporting information Table S2) were added for 30 min under the same conditions. Dead cells were excluded using eFluor780. Monoclonal antibodies for intracellular staining (Supporting information Table S1) were added after fixation and permeabilization of the cells using the Cytofix/Cytoperm Reagent Set (BD Biosciences). Cells were washed twice and analyzed on an LSRFortessa flow cytometer. The gating strategy used in the functional analysis can be found in Supporting information Fig. S3. Results from the negative controls (medium alone) are displayed in Supporting information Fig. S3. To determine the polyfunctionality of MAIT cells, the average number of functions of each MAIT cell population was calculated using the following formula: (([percentage of cells producing 1 cytokine]*1) + ([percentage of cells producing 2 cytokines]*2) + ([percentage of cells producing 3 cytokines]*3) + ([percentage of cells producing 4 cytokines]*4) + ([percentage of cells producing all 5 cytokines]*5))/100.

### Data analysis

Renal MAIT cells from the kidney samples (both transplant and healthy) were compared to the circulating MAIT cells from the blood samples (healthy controls and 1‐year post‐transplantation). Only the post‐transplantation samples were included in this analysis to avoid including each RTRs twice. Furthermore, renal tissue from the transplants explanted after allograft failure was compared to healthy renal tissue. To determine the impact of ESRD on circulating MAIT cells, pretransplantation blood samples were compared to blood samples from healthy controls. To assess the effect of the transplant and transplantation procedure, pretransplantation blood samples were compared to the 12‐month post‐transplantation samples. Data were analyzed using FlowJo version 10 (FlowJo, Ashland, OR, USA). All graphs and figures were created using Graphpad Prism version 8.00 for Windows (GraphPad Software, La Jolla, CA, USA). Data were analyzed for statistical significance using IBM's SPSS software version 23. The normality of the distribution was evaluated for each variable. For positive, right‐skewed variables, the data were log transformed and then checked for improvement. Since most variables remained non‐normally distributed, the nonparametric Mann Whitney‐U test was used to determine significance. To compare paired samples, either the Wilcoxon signed rank test (comparison of two groups) or the Friedman test (comparison of three groups) was used. *p*‐values <0.05 were considered statistically significant.

## Author contribution

M.L.T., E.B.M.R., M.C.A., and F.J.B. designed the study, M.J.S., E.B.M.R., N.D.B.B., and J.K. collected patient material, M.L.T., E.B.M.R, M.J.S., and N.D.B.B. carried out the experiments, M.L.T., M.J.S., and E.B.M.R. analyzed the data, M.L.T. and E.B.M.R. made the figures, all authors were involved in the (clinical) interpretation and explanation of the results, M.L.T. wrote the manuscript, under supervision from F.J.B, M.C.A., and S.E.G. All authors approved the final version of the manuscripts.

## Conflict of interest

Pharma. F.J.B. received an unrestricted grant from Astellas Pharma. S.E.G. received grants from NordicPharma and the Vifor Pharma group for her contribution as a consultant on (inter)national advisory boards for fosfomycin iv, temocillin and OM‐89. The others authors declare no commercial or financial conflict of interest.

**Abbreviations**
DN: double negative
Eomes: eomesdermosin
ESRD: end‐stage renal disease
GM‐CSF: granulocyte‐macrophage colony‐stimulating factor
IL‐7Rα: interleukin‐7 receptor α‐chain
KLRG‐1: killer cell lectin‐like receptor subfamily G member 1
MAIT cells: mucosal‐associated invariant T‐cells
MNCs: mononuclear cells
RTRs: renal transplant recipients
T‐bet: T‐box transcription factor
UTIs: urinary tract infections

## Supporting information

Supporting information

## References

[1] Dusseaux, M. , Martin, E. , Serriari, N. , Peguillet, I. , Premel, V. , Louis, D. , Milder, M. et al., Human MAIT cells are xenobiotic‐resistant, tissue‐targeted, CD161hi IL‐17‐secreting T cells. Blood 2011 117: 1250–1259. PMID:21084709 21084709 10.1182/blood-2010-08-303339

[2] Hinks, T. S. , Mucosal‐associated invariant T cells in autoimmunity, immune‐mediated diseases and airways disease. Immunology 2016 148: 1–12. PMID:26778581 26778581 10.1111/imm.12582 PMC4819138

[3] Booth, J. S. , Salerno‐Goncalves, R. , Blanchard, T. G. , Patil, S. A. , Kader, H. A. , Safta, A. M. , Morningstar, L. M. et al., Mucosal‐associated invariant T cells in the human gastric mucosa and blood: Role in *Helicobacter pylori* infection. Front Immunol. 2015 6: 466 PMID:26441971 26441971 10.3389/fimmu.2015.00466 PMC4585133

[4] Gibbs, A. , Leeansyah, E. , Introini, A. , Paquin‐Proulx, D. , Hasselrot, K. , Andersson, E. , Broliden, K. et al., MAIT cells reside in the female genital mucosa and are biased towards IL‐17 and IL‐22 production in response to bacterial stimulation. Mucosal Immunol. 2017 10: 35–45. PMID:27049062 27049062 10.1038/mi.2016.30 PMC5053908

[5] Law, B. M. P. , Wilkinson, R. , Wang, X. , Kildey, K. , Giuliani, K. , Beagley, K. W. , Ungerer, J. et al., Human tissue‐resident mucosal‐associated invariant T (MAIT) cells in renal fibrosis and CKD. J. Am. Soc. Nephrol. 2019 30: 1322–1335. PMID:31186283 31186283 10.1681/ASN.2018101064 PMC6622420

[6] Gherardin, N. A. , Souter, M. N. , Koay, H. F. , Mangas, K. M. , Seemann, T. , Stinear, T. P. , Eckle, S. B. et al., Human blood MAIT cell subsets defined using MR1 tetramers. Immunol. Cell Biol. 2018 96: 507–525. PMID:29437263 29437263 10.1111/imcb.12021 PMC6446826

[7] Brozova, J. , Karlova, I. and Novak, J. , Analysis of the phenotype and function of the subpopulations of mucosal‐associated invariant T cells. Scand J. Immunol. 2016 84: 245–251. PMID:27474379 27474379 10.1111/sji.12467

[8] Gold, M. C. , Cerri, S. , Smyk‐Pearson, S. , Cansler, M. E. , Vogt, T. M. , Delepine, J. , Winata, E. et al., Human mucosal associated invariant T cells detect bacterially infected cells. PLoS Biol. 2010 8: e1000407 PMID:20613858 20613858 10.1371/journal.pbio.1000407 PMC2893946

[9] Le Bourhis, L. , Martin, E. , Peguillet, I. , Guihot, A. , Froux, N. , Core, M. , Levy, E. et al., Antimicrobial activity of mucosal‐associated invariant T cells. Nat. Immunol. 2010 11: 701–708. PMID:20581831 20581831 10.1038/ni.1890

[10] Treiner, E. , Duban, L. , Bahram, S. , Radosavljevic, M. , Wanner, V. , Tilloy, F. , Affaticati, P. et al., Selection of evolutionarily conserved mucosal‐associated invariant T cells by MR1. Nature 2003 422: 164–169. PMID:12634786 12634786 10.1038/nature01433

[11] Ussher, J. E. , Bilton, M. , Attwod, E. , Shadwell, J. , Richardson, R. , de Lara, C. , Mettke, E. et al., CD161^++^ CD8^+^ T cells, including the MAIT cell subset, are specifically activated by IL‐12+IL‐18 in a TCR‐independent manner. Eur. J. Immunol. 2014 44: 195–203. PMID:24019201 24019201 10.1002/eji.201343509 PMC3947164

[12] Leeansyah, E. , Svard, J. , Dias, J. , Buggert, M. , Nystrom, J. , Quigley, M. F. , Moll, M. et al., Arming of MAIT cell cytolytic antimicrobial activity is induced by IL‐7 and defective in HIV‐1 infection. PLoS Pathog. 2015 11: e1005072 PMID:26295709 26295709 10.1371/journal.ppat.1005072 PMC4546682

[13] Kjer‐Nielsen, L. , Patel, O. , Corbett, A. J. , Le Nours, J. , Meehan, B. , Liu, L. , Bhati, M. et al., MR1 presents microbial vitamin B metabolites to MAIT cells. Nature 2012 491: 717–723. PMID:23051753 23051753 10.1038/nature11605

[14] Freeman, M. L. , Morris, S. R. and Lederman, M. M. , CD161 Expression on mucosa‐associated invariant T cells is reduced in HIV‐infected subjects undergoing antiretroviral therapy who do not recover CD4(+) T cells. Pathog. Immun. 2017 2: 335–351. PMID:28868514 28868514 10.20411/pai.v2i3.136 PMC5578469

[15] Reantragoon, R. , Corbett, A. J. , Sakala, I. G. , Gherardin, N. A. , Furness, J. B. , Chen, Z. , Eckle, S. B. et al., Antigen‐loaded MR1 tetramers define T cell receptor heterogeneity in mucosal‐associated invariant T cells. J. Exp. Med. 2013 210: 2305–2320. PMID:24101382 24101382 10.1084/jem.20130958 PMC3804952

[16] Corbett, A. J. , Eckle, S. B. , Birkinshaw, R. W. , Liu, L. , Patel, O. , Mahony, J. , Chen, Z. et al., T‐cell activation by transitory neo‐antigens derived from distinct microbial pathways. Nature 2014 509: 361–365. PMID:24695216 24695216 10.1038/nature13160

[17] Kurioka, A. , Ussher, J. E. , Cosgrove, C. , Clough, C. , Fergusson, J. R. , Smith, K. , Kang, Y. H. et al., MAIT cells are licensed through granzyme exchange to kill bacterially sensitized targets. Mucosal Immunol. 2015 8: 429–440. PMID:25269706 25269706 10.1038/mi.2014.81 PMC4288950

[18] Wang, H. , D'Souza, C. , Lim, X. Y. , Kostenko, L. , Pediongco, T. J. , Eckle, S. B. G. , Meehan, B. S. et al., MAIT cells protect against pulmonary *Legionella longbeachae* infection. Nat. Commun. 2018 9: 3350 PMID:30135490 30135490 10.1038/s41467-018-05202-8 PMC6105587

[19] Cui, Y. , Franciszkiewicz, K. , Mburu, Y. K. , Mondot, S. , Le Bourhis, L. , Premel, V. , Martin, E. et al., Mucosal‐associated invariant T cell‐rich congenic mouse strain allows functional evaluation. J. Clin. Invest. 2015 125: 4171–4185. PMID:26524590 26524590 10.1172/JCI82424 PMC4639991

[20] Foxman, B. and Brown, P. , Epidemiology of urinary tract infections: transmission and risk factors, incidence, and costs. Infect. Dis. Clin. North Am. 2003 17: 227–241. PMID:12848468 12848468 10.1016/s0891-5520(03)00005-9

[21] Hooton, T. M. , Clinical practice. Uncomplicated urinary tract infection. N. Engl. J. Med. 2012 366: 1028–1037. PMID:22417256 22417256 10.1056/NEJMcp1104429

[22] Naik, A. S. , Dharnidharka, V. R. , Schnitzler, M. A. , Brennan, D. C. , Segev, D. L. , Axelrod, D. , Xiao, H. et al., Clinical and economic consequences of first‐year urinary tract infections, sepsis, and pneumonia in contemporary kidney transplantation practice. Transpl. Int. 2016 29: 241–252. PMID:26563524 26563524 10.1111/tri.12711 PMC4805426

[23] Fishman, J. A. , Infection in solid‐organ transplant recipients. N. Engl. J. Med. 2007 357: 2601–2614. PMID:18094380 18094380 10.1056/NEJMra064928

[24] Baron, M. , Belo, R. , Cathelin, D. , Moreira‐Teixeira, L. , Cartery, C. , Rondeau, E. , Mesnard, L. et al., Innate‐like and conventional T cell populations from hemodialyzed and kidney transplanted patients are equally compromised. PLoS One. 2014 9: e105422 PMID:25144742 25144742 10.1371/journal.pone.0105422 PMC4140778

[25] Juno, J. A. , Waruk, J. L. M. , Wragg, K. M. , Mesa, C. , Lopez, C. , Bueti, J. , Kent, S. J. et al., Mucosal‐associated invariant T cells are depleted and exhibit altered chemokine receptor expression and elevated granulocyte macrophage‐colony stimulating factor production during end‐stage renal disease. Front Immunol. 2018 9: 1076 PMID:29868028 29868028 10.3389/fimmu.2018.01076 PMC5967229

[26] Betjes, M. G. , Meijers, R. W. and Litjens, N. H. , Loss of renal function causes premature aging of the immune system. Blood Purif. 2013 36: 173–178. PMID:24496187 24496187 10.1159/000356084

[27] Kato, S. , Chmielewski, M. , Honda, H. , Pecoits‐Filho, R. , Matsuo, S. , Yuzawa, Y. , Tranaeus, A. et al., Aspects of immune dysfunction in end‐stage renal disease. Clin. J. Am. Soc. Nephrol. 2008 3: 1526–1533. PMID:18701615 18701615 10.2215/CJN.00950208 PMC4571158

[28] Martin, E. , Treiner, E. , Duban, L. , Guerri, L. , Laude, H. , Toly, C. , Premel, V. et al., Stepwise development of MAIT cells in mouse and human. PLoS Biol. 2009 7: e54 PMID:19278296 19278296 10.1371/journal.pbio.1000054 PMC2653554

[29] Behr, F. M. , Chuwonpad, A. , Stark, R. and van Gisbergen, K. , Armed and ready: Transcriptional regulation of tissue‐resident memory CD8 T cells. Front Immunol. 2018 9: 1770 PMID:30131803 30131803 10.3389/fimmu.2018.01770 PMC6090154

[30] Salio, M. , Gasser, O. , Gonzalez‐Lopez, C. , Martens, A. , Veerapen, N. , Gileadi, U. , Verter, J. G. et al., Activation of human mucosal‐associated invariant T cells induces CD40L‐dependent maturation of monocyte‐derived and primary dendritic cells. J. Immunol. 2017 199: 2631–2638. PMID:28877992 28877992 10.4049/jimmunol.1700615 PMC5632842

[31] Romero, P. , Zippelius, A. , Kurth, I. , Pittet, M. J. , Touvrey, C. , Iancu, E. M. , Corthesy, P. et al., Four functionally distinct populations of human effector‐memory CD8^+^ T lymphocytes. J. Immunol. 2007 178: 4112–4119. PMID:17371966 17371966 10.4049/jimmunol.178.7.4112

[32] Hamann, D. , Baars, P. A. , Rep, M. H. , Hooibrink, B. , Kerkhof‐Garde, S. R. , Klein, M. R. and van Lier, R. A. , Phenotypic and functional separation of memory and effector human CD8^+^ T cells. J. Exp. Med. 1997 186: 1407–1418. PMID:9348298 9348298 10.1084/jem.186.9.1407 PMC2199103

[33] Appay, V. , Dunbar, P. R. , Callan, M. , Klenerman, P. , Gillespie, G. M. , Papagno, L. , Ogg, G. S. et al., Memory CD8^+^ T cells vary in differentiation phenotype in different persistent virus infections. Nat. Med. 2002 8: 379–385. PMID:11927944 11927944 10.1038/nm0402-379

[34] van Aalderen, M. C. , Remmerswaal, E. B. , Verstegen, N. J. , Hombrink, P. , ten Brinke, A. , Pircher, H. , Kootstra, N. A. et al., Infection history determines the differentiation state of human CD8^+^ T cells. J. Virol. 2015 89: 5110–5123. PMID:25717102 25717102 10.1128/JVI.03478-14 PMC4403462

[35] Greenberg, S. A. , Pinkus, J. L. , Kong, S. W. , Baecher‐Allan, C. , Amato, A. A. and Dorfman, D. M. , Highly differentiated cytotoxic T cells in inclusion body myositis. Brain 2019 142: 2590–2604. 31326977 10.1093/brain/awz207

[36] Schwartzkopff, S. , Woyciechowski, S. , Aichele, U. , Flecken, T. , Zhang, N. , Thimme, R. Pircher, H. , TGF‐beta downregulates KLRG1 expression in mouse and human CD8(+) T cells. Eur. J. Immunol. 2015 45: 2212–2217. PMID:26014037 26014037 10.1002/eji.201545634

[37] Stein, J. V. and Nombela‐Arrieta, C. , Chemokine control of lymphocyte trafficking: a general overview. Immunology 2005 116: 1–12. PMID:16108812 16108812 10.1111/j.1365-2567.2005.02183.x PMC1802414

[38] Oldham, K. A. , Parsonage, G. , Bhatt, R. I. , Wallace, D. M. , Deshmukh, N. , Chaudhri, S. , Adams, D. H. et al., T lymphocyte recruitment into renal cell carcinoma tissue: a role for chemokine receptors CXCR3, CXCR6, CCR5, and CCR6. Eur. Urol. 2012 61: 385–394. PMID:22079021 22079021 10.1016/j.eururo.2011.10.035

[39] Hoffmann, U. , Segerer, S. , Rummele, P. , Kruger, B. , Pietrzyk, M. , Hofstadter, F. , Banas, B. et al., Expression of the chemokine receptor CXCR3 in human renal allografts—a prospective study. Nephrol. Dial. Transplant. 2006 21: 1373–1381. PMID:16421159 16421159 10.1093/ndt/gfk075

[40] Nastase, M. V. , Zeng‐Brouwers, J. , Beckmann, J. , Tredup, C. , Christen, U. , Radeke, H. H. , Wygrecka, M. et al., Biglycan, a novel trigger of Th1 and Th17 cell recruitment into the kidney. Matrix Biol. 2018 68–69: 293–317. PMID:29253517 10.1016/j.matbio.2017.12.002 29253517

[41] Chung, A. C. and Lan, H. Y. , Chemokines in renal injury. J. Am. Soc. Nephrol. 2011 22: 802–809. PMID:21474561 21474561 10.1681/ASN.2010050510

[42] Scholzen, T. and Gerdes, J. , The Ki‐67 protein: from the known and the unknown. J. Cell Physiol. 2000 182: 311–322. PMID:10653597 10653597 10.1002/(SICI)1097-4652(200003)182:3<311::AID-JCP1>3.0.CO;2-9

[43] Kumar, B. V. , Ma, W. , Miron, M. , Granot, T. , Guyer, R. S. , Carpenter, D. J. , Senda, T. et al., Human tissue‐resident memory T cells are defined by core transcriptional and functional signatures in lymphoid and mucosal sites. Cell Rep. 2017 20: 2921–2934. PMID:28930685 28930685 10.1016/j.celrep.2017.08.078 PMC5646692

[44] Smolders, J. , Heutinck, K. M. , Fransen, N. L. , Remmerswaal, E. B. M. , Hombrink, P. , Ten Berge, I. J. M. , van Lier, R. A. W. et al., Tissue‐resident memory T cells populate the human brain. Nat. Commun. 2018 9: 4593 PMID:30389931 30389931 10.1038/s41467-018-07053-9 PMC6214977

[45] Clark, R. A. , Chong, B. , Mirchandani, N. , Brinster, N. K. , Yamanaka, K. , Dowgiert, R. K. and Kupper, T. S. , The vast majority of CLA+ T cells are resident in normal skin. J. Immunol. 2006 176: 4431–4439. PMID:16547281 16547281 10.4049/jimmunol.176.7.4431

[46] Hombrink, P. , Helbig, C. , Backer, R. A. , Piet, B. , Oja, A. E. , Stark, R. , Brasser, G. et al., Programs for the persistence, vigilance and control of human CD8(+) lung‐resident memory T cells. Nat. Immunol. 2016 17: 1467–1478. PMID:27776108 27776108 10.1038/ni.3589

[47] Lamoreaux, L. , Roederer, M. and Koup, R. , Intracellular cytokine optimization and standard operating procedure. Nat. Protoc. 2006 1: 1507–1516. PMID:17406442 17406442 10.1038/nprot.2006.268

[48] Dias, J. , Boulouis, C. , Gorin, J. B. , van den Biggelaar, R. , Lal, K. G. , Gibbs, A. , Loh, L. et al., The CD4(‐)CD8(‐) MAIT cell subpopulation is a functionally distinct subset developmentally related to the main CD8(+) MAIT cell pool. Proc. Natl. Acad. Sci. USA 2018 115: E11513‐E11522. PMID:30442667 30442667 10.1073/pnas.1812273115 PMC6298106

[49] Fernandez, C. S. , Amarasena, T. , Kelleher, A. D. , Rossjohn, J. , McCluskey, J. , Godfrey, D. I. and Kent, S. J. , MAIT cells are depleted early but retain functional cytokine expression in HIV infection. Immunol. Cell Biol. 2015 93: 177–188. PMID:25348935 25348935 10.1038/icb.2014.91

[50] Pallett, L. J. , Davies, J. , Colbeck, E. J. , Robertson, F. , Hansi, N. , Easom, N. J. W. , Burton, A. R. et al., IL‐2(high) tissue‐resident T cells in the human liver: sentinels for hepatotropic infection. J. Exp. Med. 2017 214: 1567–1580. PMID:28526759 28526759 10.1084/jem.20162115 PMC5461007

[51] Oo, Y. H. , Shetty, S. and Adams, D. H. , The role of chemokines in the recruitment of lymphocytes to the liver. Dig. Dis. 2010 28: 31–44. PMID:20460888 20460888 10.1159/000282062 PMC2883834

[52] Sobkowiak, M. J. , Davanian, H. , Heymann, R. , Gibbs, A. , Emgard, J. , Dias, J. , Aleman, S. et al., Tissue‐resident MAIT cell populations in human oral mucosa exhibit an activated profile and produce IL‐17. Eur. J. Immunol. 2019 49: 133–143. PMID:30372518 30372518 10.1002/eji.201847759 PMC6519349

[53] Weaver, C. T. , Elson, C. O. , Fouser, L. A. and Kolls, J. K. , The Th17 pathway and inflammatory diseases of the intestines, lungs, and skin. Annu. Rev. Pathol. 2013 8: 477–512. PMID:23157335 23157335 10.1146/annurev-pathol-011110-130318 PMC3965671

[54] Bamias, G. , Arseneau, K. O. and Cominelli, F. , Cytokines and mucosal immunity. Curr. Opin. Gastroenterol. 2014 30: 547–552. PMID:25203451 25203451 10.1097/MOG.0000000000000118 PMC4234041

[55] Flannigan, K. L. , Ngo, V. L. , Geem, D. , Harusato, A. , Hirota, S. A. , Parkos, C. A. , Lukacs, N. W. et al., IL‐17A‐mediated neutrophil recruitment limits expansion of segmented filamentous bacteria. Mucosal Immunol. 2017 10: 673–684. PMID:27624780 27624780 10.1038/mi.2016.80 PMC5350071

[56] Guglani, L. and Khader, S. A. , Th17 cytokines in mucosal immunity and inflammation. Curr. Opin HIV AIDS 2010 5: 120–127. PMID:20543588 20543588 10.1097/COH.0b013e328335c2f6 PMC2892849

[57] Muruganandah, V. , Sathkumara, H. D. , Navarro, S. and Kupz, A. , A systematic review: the role of resident memory T cells in infectious diseases and their relevance for vaccine development. Front Immunol. 2018 9: 1574 PMID:30038624 30038624 10.3389/fimmu.2018.01574 PMC6046459

[58] Masopust, D. and Soerens , A. G. , Tissue‐resident T cells and other resident leukocytes. Annu. Rev. Immunol. 2019 37: 521–546. PMID:30726153 30726153 10.1146/annurev-immunol-042617-053214 PMC7175802

[59] Dandrieux, J. R. S. , Narayanan, L. , Firestone, S. , Archer, T. M. and Mansfield, C. S. , Effect of immunosuppressive drugs on cytokine production in canine whole blood stimulated with lipopolysaccharide or a combination of ionomycin and phorbol 12‐myristate 13‐acetate. Vet. Med. Sci. 2019 5: 199–205. PMID:30663866 30663866 10.1002/vms3.143 PMC6498811

[60] Cossarizza, A. , Chang, H. D. , Radbruch, A. , Acs, A. , Adam, D. , Adam‐Klages, S. , Agace, W. W. et al., Guidelines for the use of flow cytometry and cell sorting in immunological studies (second edition). Eur. J. Immunol. 2019 49: 1457–1973. PMID:31633216 31633216 10.1002/eji.201970107 PMC7350392

[61] Dias, J. , Sobkowiak, M. J. , Sandberg, J. K. and Leeansyah, E. , Human MAIT‐cell responses to Escherichia coli: activation, cytokine production, proliferation, and cytotoxicity. J. Leukoc. Biol. 2016 100: 233–240. PMID:27034405 27034405 10.1189/jlb.4TA0815-391RR PMC4946616

