# Supplementary material for: Tissue‐resident mucosal‐associated invariant T (MAIT) cells in the human kidney represent a functionally distinct subset
Source: Eur J Immunol. 2020 Aug 6;50(11):1783–97. doi: 10.1002/eji.202048644 (PMC7689767; doi:10.1002/eji.202048644)
Supplement: Supplementary file 1 — Supporting information [file EJI-50-1783-s001.pdf]

Table of Content Supplemental Material

Supplemental Table 1. Monoclonal antibodies used for phenotyping.

Supplemental Table 2. Monoclonal antibodies used for stimulation assay.

Supplemental Figure 1. Gating strategy for phenotypical analysis of MAIT cells in PBMCs and kidney MNCs.

Supplemental Figure 2. Enzymatic digestion affects CCR6-expression; all other markers tested are unaltered.

Supplemental Figure 3. Gating strategy for functional analysis of MAIT cells and CD3+CD8+ T cells in PBMCs and kidney MNCs after stimulation with PMA and Ionomycin.

Supplemental Figure 4. CD3- and MAIT cell counts.

Supplemental Figure 5. Absolute CD3 and MAIT cell numbers.

Supplemental Figure 6. Polyfunctional analysis of MAIT cells.

Supplemental Figure 7. Polyfunctional analysis of CD103- and CD103+ MAIT cells in kidney MNCs.

Supplemental Table 1. Monoclonal antibodies used for phenotyping.

anti	clone	fluorochrome	staining	manufacturer
CD3	UCHT1	BUV496	surface	BD Bioscience[1]
CD3	UCHT1	V500	surface	BD Bioscience[1]
CD4	SK3	BUV563	surface	BD Bioscience[1]
CD4	RPA-T4	APC-R700	surface	BD Bioscience[1]
CD4	RPA-T4	BV650	surface	BD Bioscience[1]
CD4	SK3	PerCP-eFluor 710	surface	eBioscience Inc.[2]
CD103	Ber-ACT8	BUV395	surface	BD Bioscience[1]
CD103	Ber-ACT8	BV711	surface	BD Bioscience[1]
CD161	DX12	PE	surface	BD Bioscience[1]
CD161	DX12	FITC	surface	BD Bioscience[1]
CD45	HI30	PerCP-eFluor 710	surface	eBioscience Inc.[2]
CD45RA	HI100	BV650	surface	BD Bioscience[1]
CD197 (CCR7)	150503	BUV395	surface	BD Bioscience[1]
CD197 (CCR7)	2-L1-A	PE-CF594	surface	BD Bioscience[1]
CD279 (PD1)	EH12	BB515	surface	BD Bioscience[1]
CD127 (IL7R-α)	eBioRDR5	PE-Cy7	surface	eBioscience Inc.[2]
CD4	SK3	PerCP-eFluor 710	surface	eBioscience Inc.[2]
CD8	RPA-T8	BV785	surface	Sony Biotechnology[3]
CD27	M-T271	APC	surface	BD Bioscience[1]
CD27	O323	APC-Fire750	surface	BioLegend[4]
CD28	CD28.2	APC	surface	BD Bioscience[1]
CD28	CD28.2	APC-R700	surface	BD Bioscience[1]
CD186 (CXCR6)	K041E5	PE-Cy7	surface	BioLegend[4]
CD196 (CCR6)	G034E3	AF488	Surface	BioLegend[4]
CD69	FN50	APC-Fire750	surface	BioLegend[4]
CD183 (CXCR3)	REA232	PE-Vio615	surface	Miltenyi Biotec[5]
KLRG1	3F12F2	AF488	surface	eBioscience Inc.[2]
KLRG1	3F12F2	PerCP-eFluor 710	surface	eBioscience Inc.[2]
Ki-67	Ki-67	BV711	intracellular	BioLegend[4]
Ki-67	Ki-67	AF700	intracellular	BD Bioscience[1]
granzyme B	GB11	AF700	intracellular	BD Bioscience[1]
T-bet	4B10	PE-Cy7	intracellular	eBioscience Inc.[2]
T-bet	4B10	BV711	intracellular	BioLegend[4]
Eomes	WD1928	eFluor 660	intracellular	eBioscience Inc.[2]

[1]Franklin Lakes, NJ, USA [2]Thermo Fisher Scientific, San Diego, CA, USA [3]Weybridge, UK
[4]San Diego, CA, USA [5]Bergisch Gladbach, Germany

Supplemental Table 2. Monoclonal antibodies used for stimulation assay.

anti	clone	fluorochrome	staining	manufacturer
CD3	SK7	PerCP-eFluor 710	surface	eBioscience Inc.[2]
CD4	RPA-T4	BV711	surface	BioLegend[4]
CD161	HP-3G10	PE-Cy7	surface	BioLegend[4]
CD8	RPA-T8	BV785	surface	Sony Biotechnology[3]
CD103	Ber-ACT8	BB515	surface	BD Bioscience1
IFNγ	B27	BUV 395	intracellular	BD Bioscience[1]
TNFα	Mab11	AF700	intracellular	BD Bioscience[1]
IL-17A	N49-653	BV650	intracellular	BD Bioscience[1]
IL-2	MQ1-17H12	BV510	intracellular	BioLegend[4]
GM-CSF	BVD2-21C11	PE-Dazzle	intracellular	BioLegend[4]

[1]Franklin Lakes, NJ, USA [2]Thermo Fisher Scientific, San Diego, CA, USA [3]Weybridge, UK [4]San Diego, CA, USA

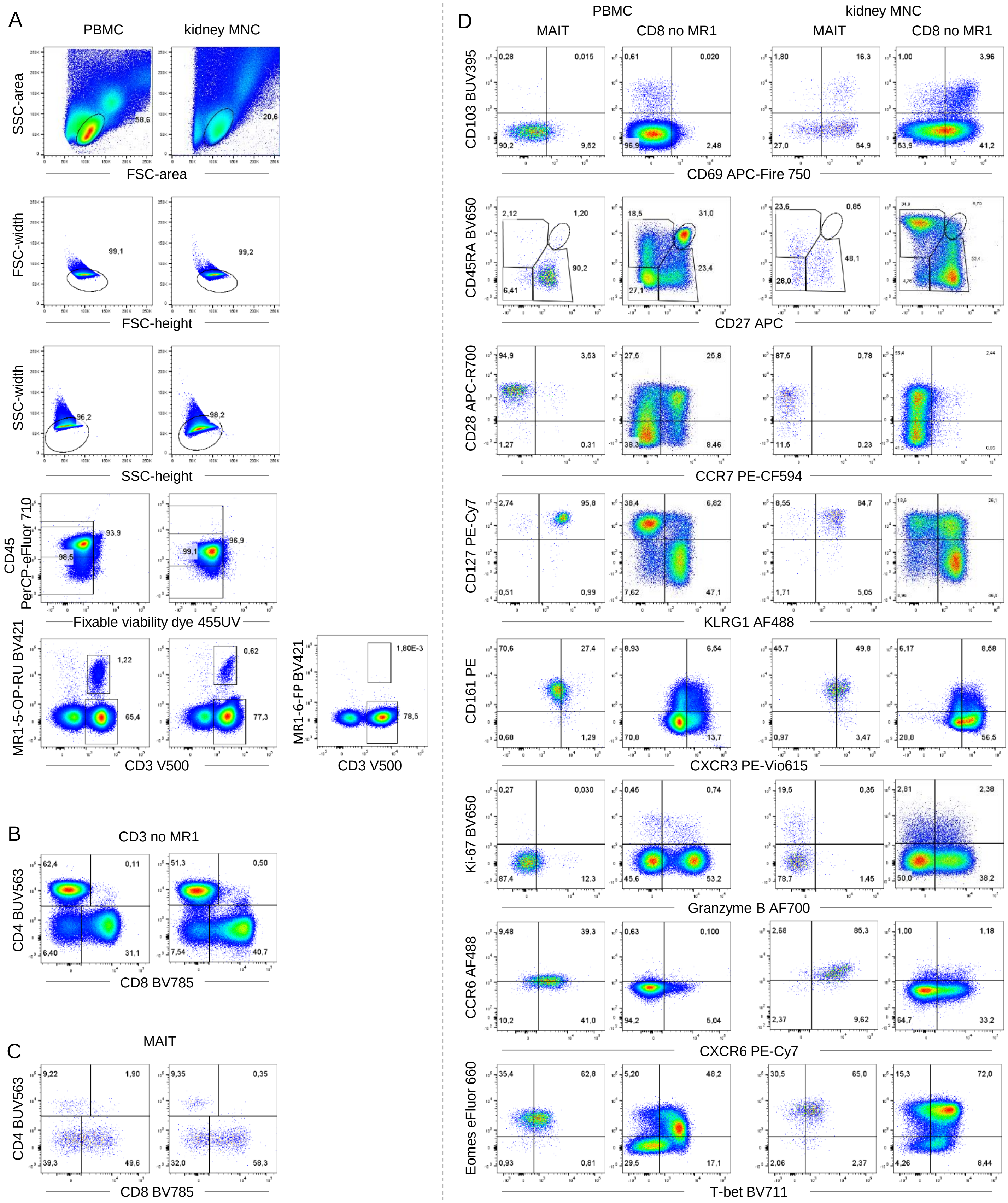

Supplemental Figure 1. Gating strategy for phenotypical analysis of MAIT cells in PBMCs and kidney MNCs. (A) MAIT cells were selected by gating MR1-5-OP-RU tetramer+CD3+CD45+live single cell lymphocytes in PBMCs (left) and kidney MNCs (right). MR1-6-FP tetramer staining is shown as a control. (B-C) CD8 and CD4 gating of (B) CD3+MR1-CD45+live single cell lymphocytes (CD3 no MR1) and (C) MAIT cells in PBMCs (left) and kidney MNCs (right). (D) Examples of gating for CD69 vs CD103, CD27 vs CD45RA, CCR7 vs CD28, CXCR3 vs CD161, granzyme B vs Ki-67, CXCR6 vs CCR6 and T-bet vs Eomes, within MAIT cells and as comparison CD3+MR1-CD8+ (CD8 no MR1) T cells in PBMCs (left) and kidney MNCs (right).

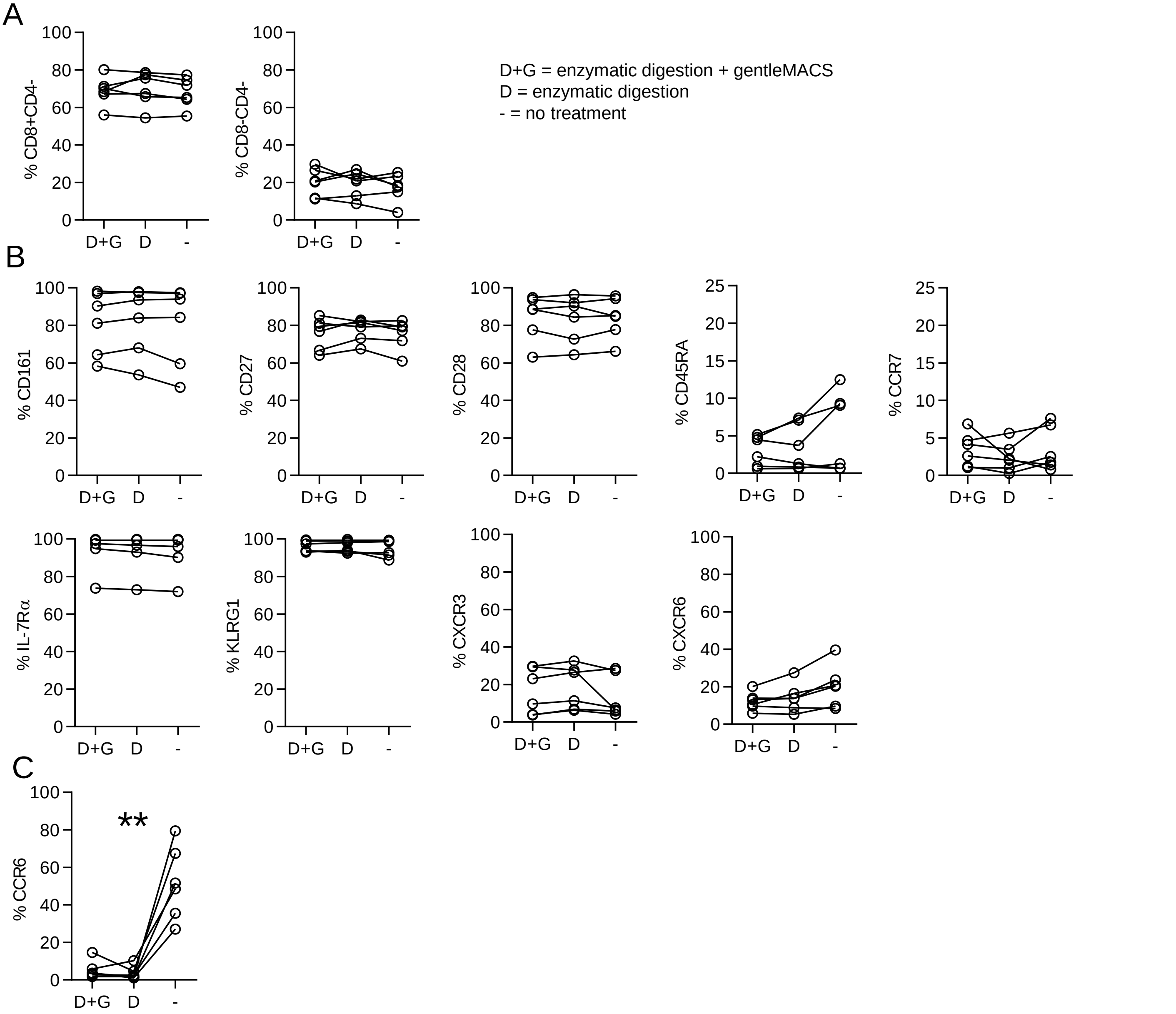

Supplemental Figure 2. Enzymatic digestion affects CCR6-expression; all other markers tested are unaltered.

In order to test the effect of the enzymatic digestion and gentleMACS treatment that was used to isolate kidney MNC on (surface) expression of all markers analyzed, freshly isolated PBMC were subjected to the enzymatic digestion followed by gentleMACS (D+G), enzymatic digestion alone (D) or no treatment at all (-), where after they where cryopreserved until the day of analysis. Flow cytometry was performed as described in the methods.

(A) Percentages of CD8+CD4- (CD8) and CD8-CD4- (DN) MAIT cells in the total MAIT cell population.

(B) Percentages of CD161, CD27, CD28, CD45RA, CCR7, IL-7Rα, KLRG1, CXCR3 and CXCR6 in CD8 MAIT cells are unaffected by enzymatic digestion or enzymatic digestion followed by gentleMACS

(C) Expression of CCR6 in CD8 MAIT cells is downregulated by digestion and digestion combined with gentleMACS.

A-C Data shown are representative of 1 independent experiments with *n* = 6 Healthy PBMCs.

The Friedman test was used to compare the three treatment protocols. Only statistical significant p-values are displayed. ** p≤0.01

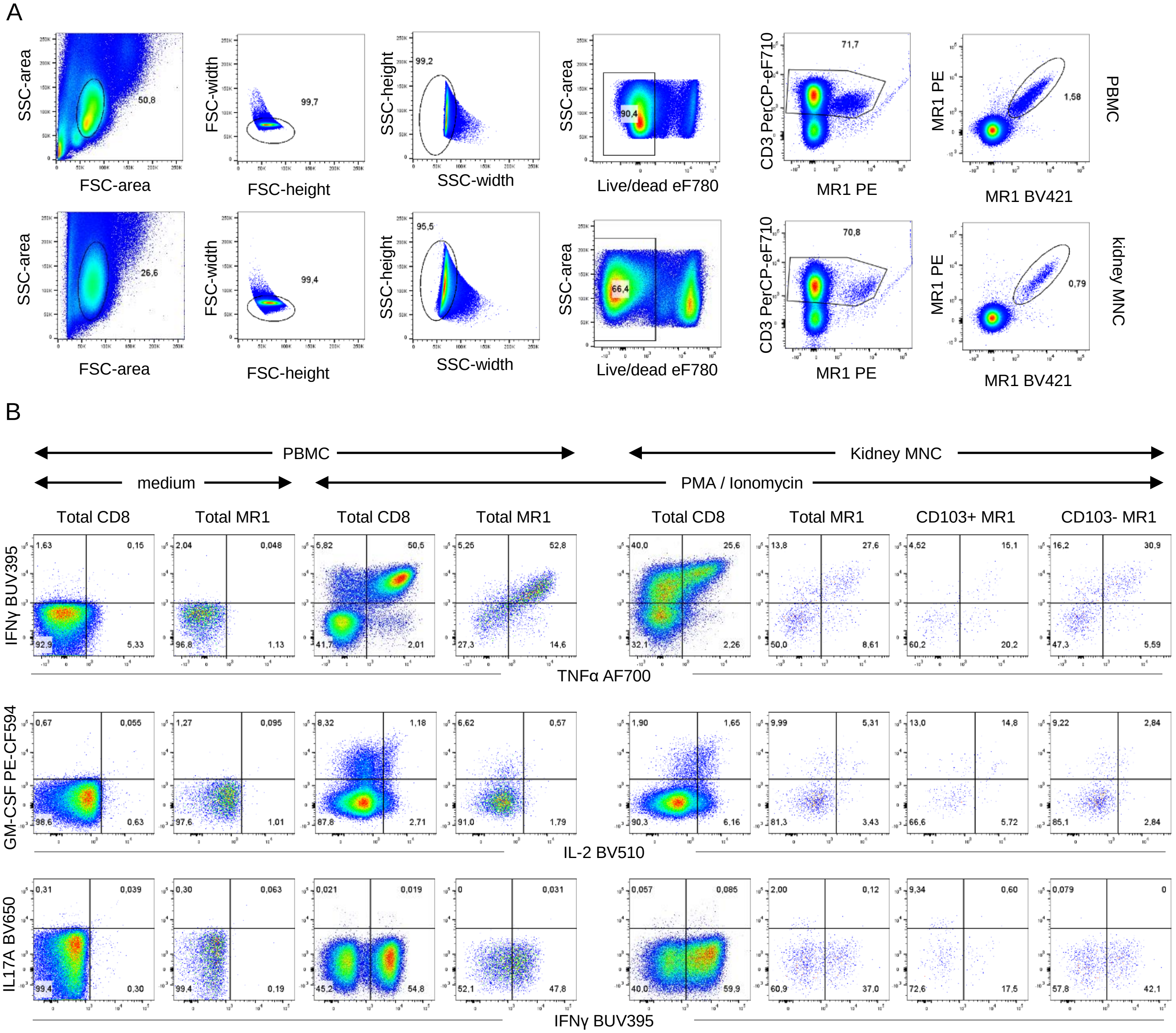

Supplemental Figure 3. Gating strategy for functional analysis of MAIT cells and CD3+CD8+ T cells in PBMCs and kidney MNCs after stimulation with PMA and Ionomycin.
(A) After stimulation with PMA and Ionomycin MAIT cells were selected by gating MR1+ cells in CD3+ live single cell lymphocytes in PBMCs (upper panel) and kidney MNCs (lower panel)
(B) Examples of gating of TNFα vs IFNγ (upper panel), IL-2 vs GM-CSF (middle panel) and IFNγ vs IL-17A (lower panel) within MAIT cells and total CD3+CD8+ cells from PBMCs (left panel) and kidney MNCs (right panel) after stimulation with PMA/ionomycin (right panel) and medium as a control (left panel).

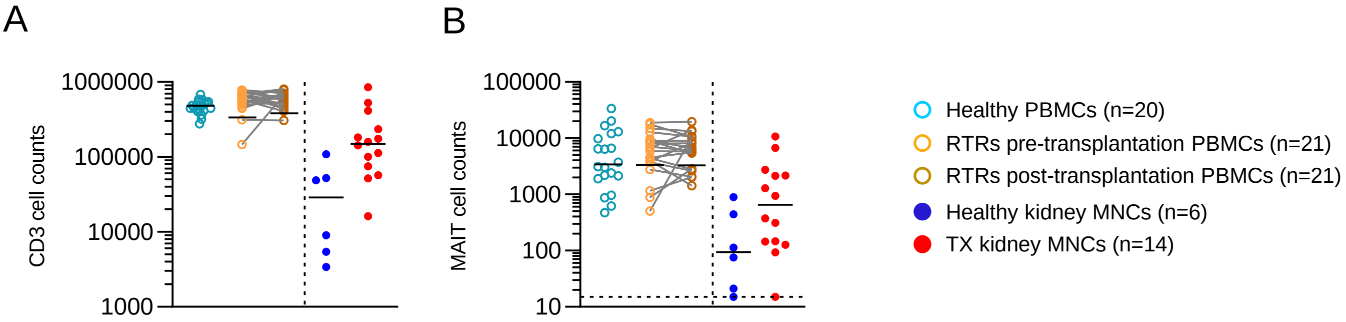

Supplemental Figure 4. CD3- and MAIT cell counts

Scatterplots of the cell counts in our analysis of (A) $CD3^+$ cells and (B) MAIT cells analyzed per sample in healthy PBMCs, RTRs pre-transplantation PBMCs, paired post-transplantation PBMCs, healthy kidney MNCs and TX kidneys MNCs. The dotted line represents the cut-off of 15 cells for further phenotyping of MAIT cells.

No statistical comparisons were made. The horizontal dash represents the median.

Data shown represent nine experiments with *n* = 2, 4, 9, 2, 21, 10, 16, 15 and 12 donors. 82 unique donors are shown.

PBMCs: peripheral blood mononuclear cells; RTRs: renal transplant recipients; MNCs: mononuclear cells; TX: transplant

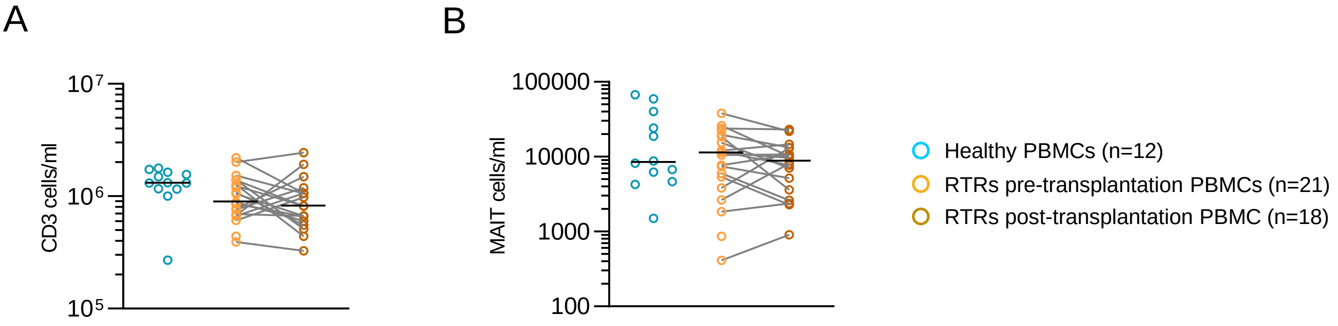

Supplemental Figure 5. Absolute CD3 and MAIT cell numbers

Absolute CD3- and MAIT cells in numbers in PBMCs. Absolute MAIT cell number were calculated from the absolute number of lymphocytes (determined by the clinical laboratory in the Amsterdam UMC, location AMC (LAKC)) with the following formula: [absolute number of lymphocytes]*[percentage of MAIT cells in live lymphocytes]/100. Absolute numbers from renal tissue were unavailable.

Scatterplots of the absolute cell numbers of (A) $CD3^+$ cells and (B) MAIT cells in healthy PBMCs, RTRs pre-transplantation PBMCs, paired post-transplantation PBMCs, healthy kidney MNCs and TX kidneys MNCs. The following statistical comparisons were made: RTRs pre-transplantation vs. healthy PBMCs (Mann Whitney U-test); RTRs pre-vs. post-transplantation PBMCs (Wilcoxon signed rank test). The horizontal dash represents the median. No significant differences were found.

Data shown represent four experiments with *n* = 21, 10, 11 and 9 donors. 51 unique individuals are shown.

PBMCs: peripheral blood mononuclear cells; RTRs: renal transplant recipients

A

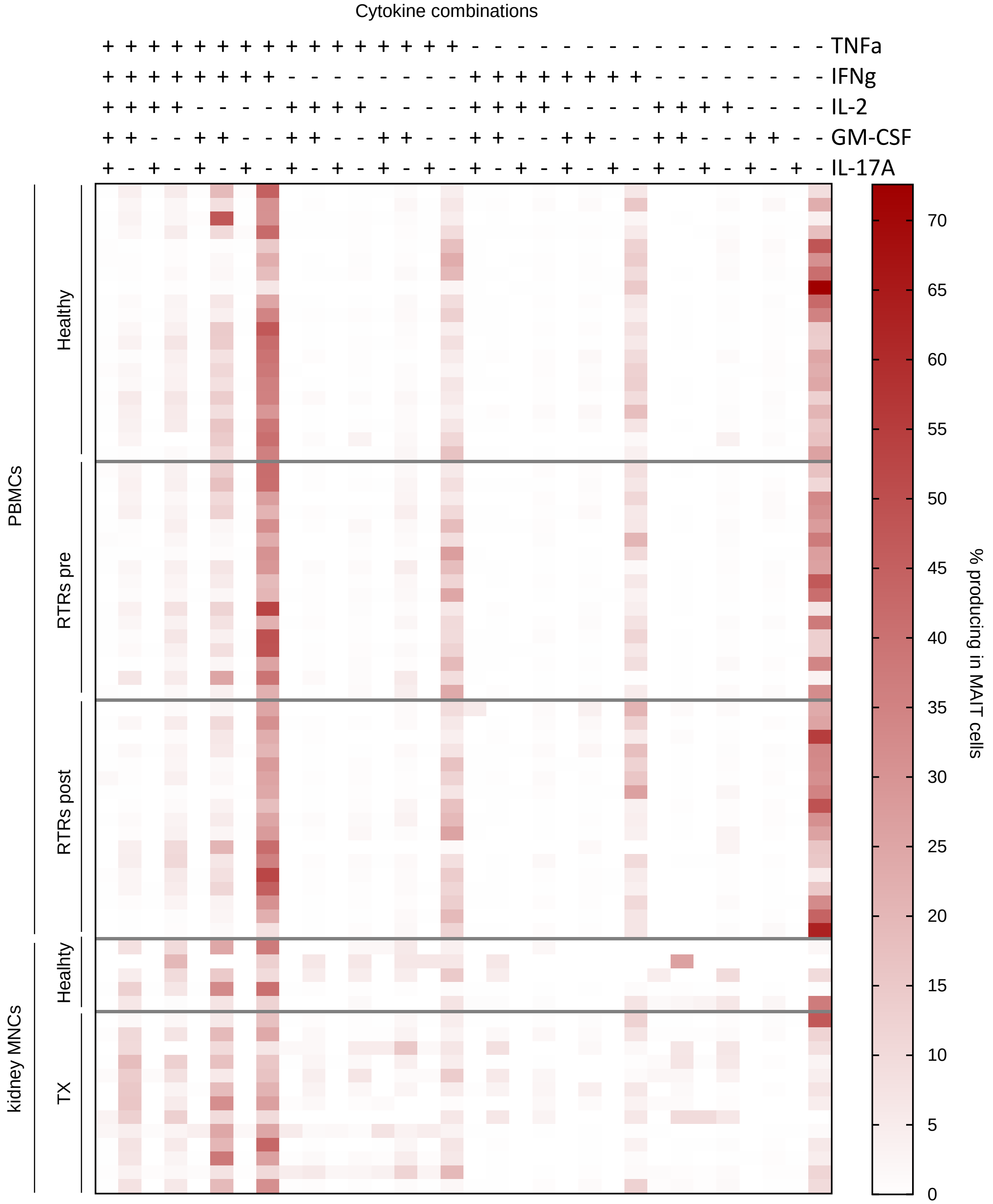

Supplemental Figure 6. Polyfunctional analysis of MAIT cells

(A) Heatmap of percentages of MAIT cells from healthy PBMCs, RTRs pre-transplantation PBMCs, paired post-transplantation PBMCs, healthy kidney MNCs and TX kidneys MNCs producing combinations of different cytokines after stimulation with PMA and Ionomycin.

Combinations of cytokines produced by at least 5% of MAIT cells (median values) in one of the study groups were further analyzed (figure 5B).

Data shown are representative of 8 independent experiments with *n* = 3, 8, 9, 10, 12, 12, 7 and 12 individuals per experiment. 72 unique individuals are shown (Healthy PBMCs= 20; RTRs pre / post transplantation PBMCs= 17, Healthy kidney MNCs = 5 and TX kidney MNCs = 13).

PBMCs: peripheral blood mononuclear cells; RTRs; renal transplant recipients; MNCs: mononuclear cells; TX: transplant

B

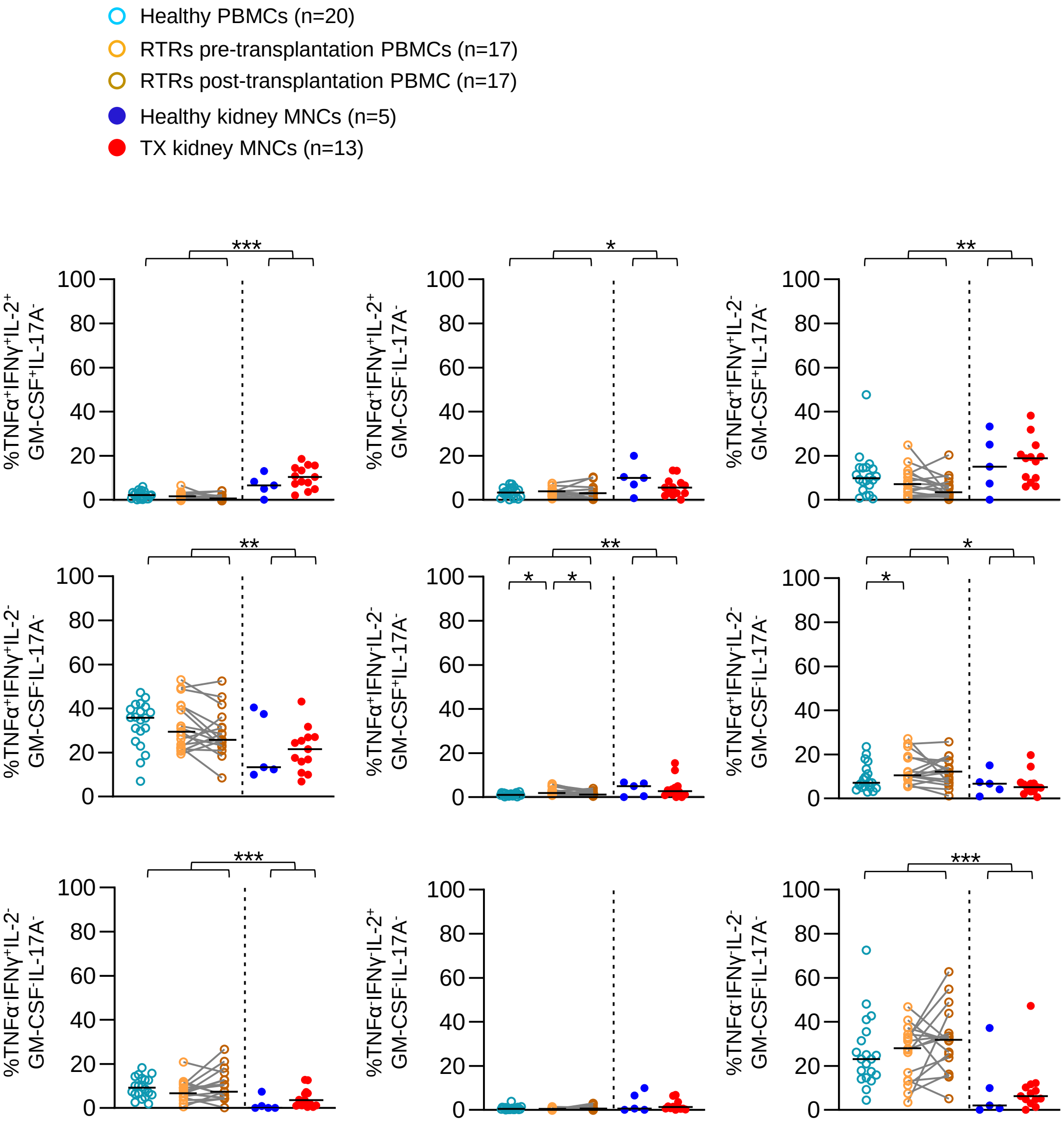

Supplemental Figure 6 continued. Polyfunctional analysis of MAIT cells
(B) Scatterplots and statistical analysis of the most frequent combinations of cytokines produced by MAIT cells.
The following statistical comparisons were made: kidney MNCs (both healthy and TX) vs. PBMCs (healthy and RTRs post-transplantation) (Mann Whitney U-test); healthy kidney vs. TX kidney MNCs (Mann Whitney U-test); RTRs pre-transplantation vs. healthy PBMCs (Mann Whitney U-test); RTRs pre-vs. post-transplantation PBMCs (Wilcoxon signed rank test). The horizontal dash represents the median. Only significant p-values are displayed: * $p < 0.05$, ** $p \leq 0.01$, *** $p \leq 0.001$.
Data shown are representative of 8 independent experiments with *n* = 3, 8, 9, 10, 12, 12, 7 and 12 individuals per experiment. 72 unique individuals are shown.

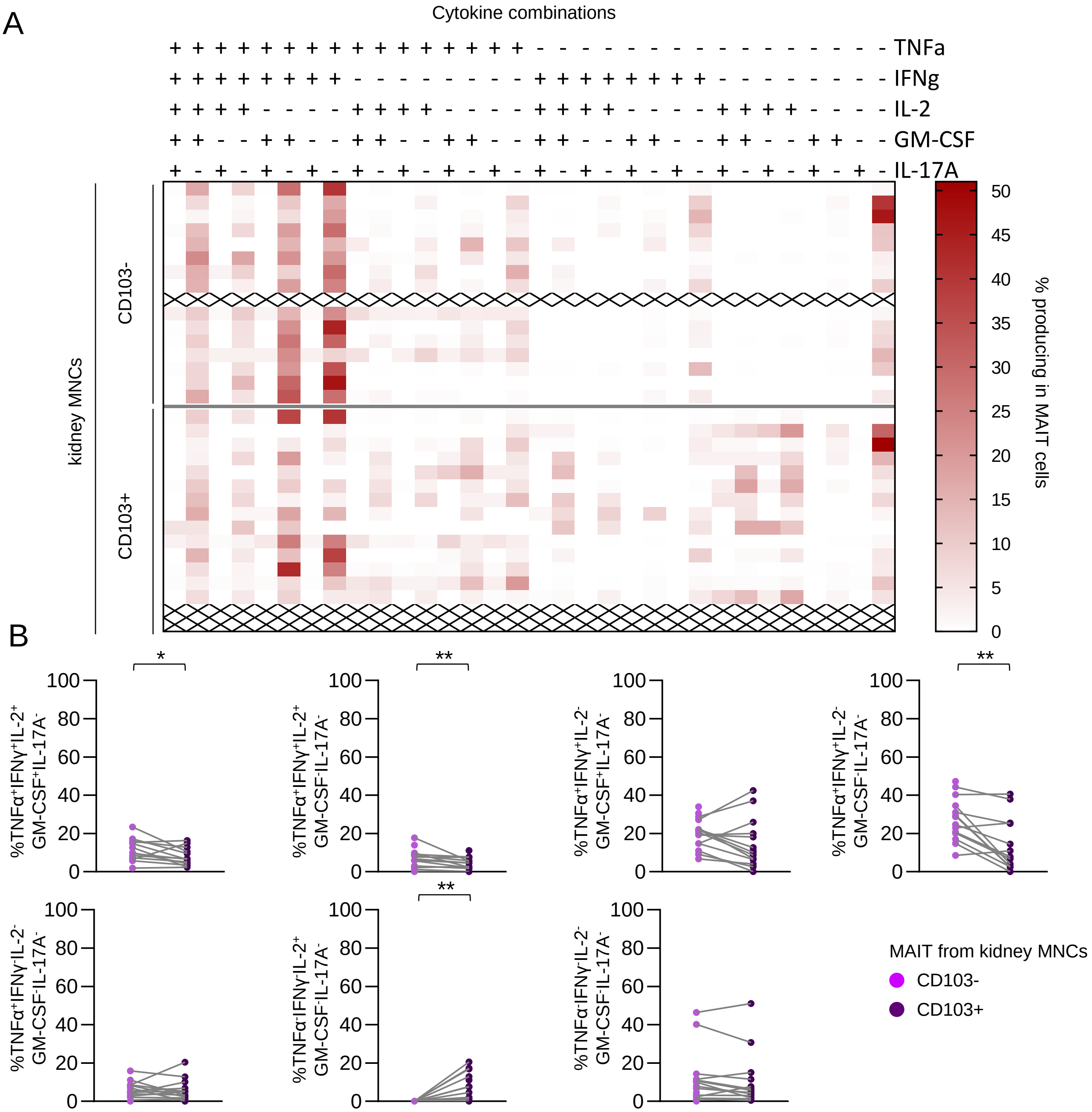

Supplemental Figure 7. Polyfunctional analysis of CD103- and CD103+ MAIT cells in kidney MNCs
Comparison of combinations of cytokines produced by CD103^{+} vs. CD103^{-} MAIT cells. The production of only IL-2 appeared to be more frequent among CD103^{+} MAIT cells, whilst production of TNFα combined with production of IFNγ was more frequent among CD103^{-} MAIT cells.
(A) Heatmap of percentages of MAIT cells producing combinations of cytokines within CD103-negative and CD103-positive MAIT cell populations from kidney MNCs after stimulation with PMA and Ionomycin. Combinations of cytokines produced by at least 5% of MAIT cells (median values) in one of the study groups were further analyzed (Supplemental figure 6B).
(B) Scatterplots and statistical analysis of the most frequent combinations of cytokines produced by CD103^{+} and CD103^{-} MAIT cells.
Wilcoxon signed rank test was used for statistical comparison. Only significant p-values are displayed: * $p<0.05$, ** $p\leq0.01$, *** $p\leq0.001$.
Data shown are representative of 2 independent experiments with n = 7 and 12 individuals per experiment. 16 unique individuals are shown (Healthy kidney MNCs = 3 and TX kidney MNCs = 13) (two samples did not contain sufficient CD103^{+} MAIT cells for analysis and one sample did not contain sufficient CD103^{-} MAIT cells for analysis). PBMCs: peripheral blood mononuclear cells; MNCs: mononuclear cells; TX: transplant
